# Molecular Diagnosis of Melanocytic Neoplasia: Updates and Challenges

**DOI:** 10.3390/cancers18203330

**Published:** 2026-10-15

**Authors:** Jaclyn M. Plotzke, Grace Wang, Scott C. Bresler, Paul W. Harms

**Affiliations:** 1 Department of Pathology, Michigan Medicine, University of Michigan, Ann Arbor, MI 48109, USA; jafurman@med.umich.edu (J.M.P.);; 2 Department of Dermatology, Michigan Medicine, University of Michigan, Ann Arbor, MI 48109, USA; 3 Rogel Cancer Center, Michigan Medicine, University of Michigan, Ann Arbor, MI 48109, USA

**Keywords:** melanoma, nevus, melanocytoma, Spitz, molecular diagnosis, fusion, fluorescence in-situ hybridization, array comparative genomic hybridization, next generation sequencing, oncogene, tumor suppressor

## Abstract

Distinction of melanoma from less aggressive melanocytic neoplasms is essential to avoid over- or undertreatment. Melanocytic neoplasms can harbor a broad spectrum of molecular drivers, including mutations, amplifications, and fusions, associated with an array of morphologies and biologic behavior. This molecular complexity creates challenges for classification and diagnosis of malignancy. Here, we review current knowledge to present a molecular framework for classifying melanocytic neoplasia and describe current molecular testing modalities useful for informing accurate diagnosis in challenging cases.

## Simple Summary

Rationale: Genomic classification of melanocytic neoplasia is of increasing importance for accurate diagnosis. Challenges to practical application of this knowledge include the complex biology of melanocytic neoplasms and nuances of diagnostic test selection. This review aims to synthesize knowledge on melanocytic genomics and outline diagnostic approaches for test selection and interpretation.Objectives are to describe: (1) Importance of diagnostic distinction of benign from malignant melanocytic tumors. (2) Mechanisms of melanocytic neoplasia initiation and progression, related to the biological categories nevus, melanocytoma, and melanoma. (3) Classes of melanocytic neoplasm, including morphologic and molecular features. (4) Molecular diagnostic test options. (5) Synthesis of current knowledge to propose optimal test panels for specific diagnostic scenariosImpact: Improved understanding of melanocytic genomic alterations and molecular assays has potential to guide more effective test selection during diagnosis of challenging cases, and will clarify knowledge gaps where research is needed.

## 1. Introduction

The spectrum of melanocytic neoplasia ranges from benign nevi to borderline/intermediate lesions to melanoma. Increasingly, molecular techniques are being used to more precisely classify entities within this spectrum. The accurate classification of a melanocytic lesion as benign versus malignant is essential for guiding management and to avoid over- and undertreatment. While melanoma is less common than basal cell carcinoma or squamous cell carcinoma, it accounts for the majority of skin-cancer-related deaths [1,2], and the global incidence continues to increase [3,4,5,6,7,8].

In this review, we detail the current molecular landscape and the appropriate use of molecular assays in classification of melanocytic neoplasia.

## 2. Overview

**Figure 1:**
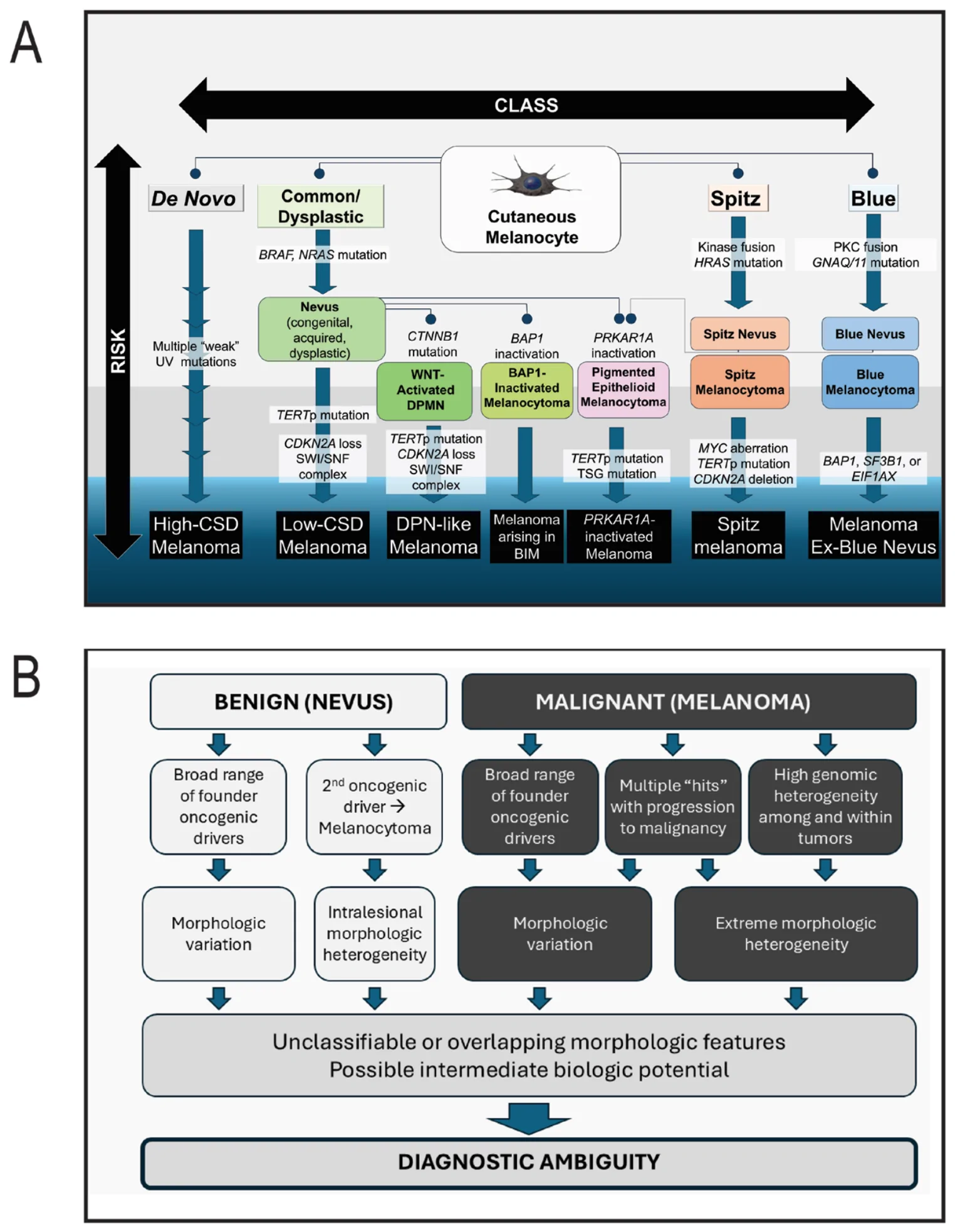
(**A**) Schematic for classification of melanocytic neoplasms. (**B**) Molecular basis for diagnostic ambiguity in melanocytic neoplasia.

Melanocytic neoplasms can be divided into three general categories defined by morphology and predicted biologic potential: nevus (benign), melanocytoma (benign to intermediate risk), and melanoma (malignant) (Figure 1A). Of note, diagnostically challenging cases may display mixed/borderline features that span these benign and malignant categories (Figure 1B) [9,10,11,12,13].

Nevi are characterized by a single driver mutation, often a strong activator of the MAPK pathway (Figure 1A). Although other mutations might be detected, in general, there is no additional strong oncogenic driver or disruption of cell cycle regulation [11,12,14].

Melanocytomas are a complex and evolving designation with a range of potential morphologic and genomic features [10,11,12,14], generally characterized by a prominent dermal component. Some classes of melanocytoma are defined by a second driver mutation superimposed upon the founder MAPK-activating mutation (Figure 1A) [11,12,14]. Other classes of melanocytoma may lack a known second driver mutation but have a dermal growth pattern more characteristic of a tumor than a nevus, characterized by increased cellularity, mitotic activity, and/or cytologic atypia [9,14]. Importantly, the biologic potential of melanocytomas is variable, and by current definitions this term should not automatically be considered synonymous with “uncertain malignant potential” [10]. Although the presence of a second oncogenic “hit” implies potential for progression, most melanocytomas are essentially benign, with an indolent course similar to a nevus [11]. However, some cases may have intermediate/indeterminate or high risk for a more aggressive course. Notably, even indolent melanocytomas have propensity for microscopic involvement of draining lymph nodes without further dissemination (so-called “benign metastasis”) [9,11]. Melanomas are the malignant end of this spectrum and display remarkable intertumoral heterogeneity with regard to oncogene and tumor suppressor inactivation events, precluding a uniformly applicable molecular definition [9].

Management of melanocytic neoplasms hinges on diagnosis. Wholly benign nevi may be monitored or excised for cosmetic purposes (with additional considerations for large congenital nevi that have increased risk for malignant progression). Complete excision is recommended for severely atypical nevi and melanocytomas [10]. Management of melanoma follows NCCN guidelines, including wide excision and sentinel lymph node mapping for deeper tumors that are clinically node-negative. Depending on the disease stage, clinical monitoring may be supplemented with imaging studies. Systemic therapies such as checkpoint inhibitors or BRAF/MEK inhibitors are in routine use for advanced melanoma, with increasing use in the neoadjuvant or adjuvant setting [11,15].

The implications of misdiagnosis relate to the aggressiveness of melanoma and potential adverse impacts of management. Misdiagnosis of a melanoma as a nevus has measurable negative impacts. In a recent large retrospective study, at least 2.3% of melanomas were initially misdiagnosed as benign. Histopathologic misdiagnosis resulted in a median delay of 29 months to reach the correct diagnosis. False negative diagnosis was associated with increased Breslow depth, higher recurrence rates, and higher rates of melanoma-specific death [16].

Misdiagnosis of a nevus as melanoma also occurs, although the population-level frequency of this error and the impact of associated morbidities is challenging to quantitate. The diagnosis of melanoma is associated with potential procedural morbidities, psychosocial impact, and financial burdens [17]; these are imposed unnecessarily if the melanocytic lesion was in fact benign. The increasing use of adjuvant immunotherapy (with potential for permanent or fatal side effects) raises the risk of serious harm to the patient if a nodal nevus or indolent nodal deposit of melanocytoma is misinterpreted as metastatic melanoma.

The implications of misdiagnosis, together with the morphologic heterogeneity of melanocytic neoplasms, underscores the need for investigations into molecular mechanisms of melanocytic neoplasia. Molecular drivers of melanocytic neoplasia are associated with distinctive morphologies that define classes of melanocytic neoplasm (Figure 1A), including conventional/dysplastic (*BRAF*, *NRAS* mutation), Spitz (kinase fusion or *HRAS* mutation), and blue (*GNAQ*/*11* activation or protein kinase C fusion) [14]. Each class is associated with subtypes of nevus, melanocytoma, and melanoma. Notably, a morphologic feature that would be concerning for malignancy if observed in one class might be an established characteristic of nevi in another class. In addition, some molecular drivers are more strongly associated with nevi or melanoma. Thus, molecular characterization of a melanocytic neoplasm can provide multiple data points regarding class and risk, facilitating more precise diagnosis [11].

## 3. Molecular Features of Melanocytic Neoplasia and Progression to Melanoma

Similar to other cell types, neoplastic transformation of a melanocyte is mediated by an imbalance of tumor suppressor and oncogene activity [11,18]. Understanding mechanisms of malignant progression has been challenging due to the extreme genomic heterogeneity of melanoma, with no single genetic alteration being totally sensitive and specific for melanoma relative to benign melanocytes. Furthermore, melanocytes are dispersed at low density among other cell types in normal skin and many nevi are relatively small, raising challenges for robust molecular comparisons with melanoma. It is often unclear whether a particular molecular difference is a driver or consequence of malignancy. Although these obstacles continue to be relevant, recent studies have substantially advanced understanding of the molecular pathways involved in the formation of melanoma [19,20,21].

Current evidence suggests that melanoma arises via one of several major mechanisms: low CSD pathways involving progression from a benign precursor (nevus), high CSD pathways involving *de novo* formation without a precursor nevus, or UV-independent pathways related to sun-protected sites, Spitz nevi, or congenital nevi (Figure 1A) [9,19,21,22].

The low-CSD pathways involve progression of a conventional nevus (or a melanocytoma arising in a conventional nevus) to melanoma. Conventional nevi are associated with strong MAPK activating mutations such as *BRAF* V600E or *NRAS* Q61R [23,24,25,26]. Founder *BRAF* mutations have traditionally been considered spontaneous rather than UV-mediated, although this has been challenged [27]. New evidence suggests a precursor field of *BRAF* mutated melanocytes may precede nevus formation [20]. During nevus formation, nevomelanocytes undergo limited proliferation before tumor suppressors trigger cell cycle arrest via senescence [28]. Second genomic hits may trigger formation of a melanocytoma that also enters growth arrest. In a small fraction of nevi or melanocytomas, additional genomic alterations result in escape from senescence and progression to melanoma. Tumor suppressor inactivation events that may be implicated in malignant progression include homozygous deletions affecting tumor suppressor genes on chromosome 9p21, especially *CDKN2A* [19]. Inactivation of the chromatin modifier SWI/SNF component genes such as *ARID1A*, *ARID1B*, and *ARID2* is also frequently observed [18,29]. *TP53* and *PTEN* mutations are less frequent and may arise later during melanoma progression [29]. *TERT* promoter (*TERT*p) mutations that circumvent replicative senescence are associated with malignancy in many contexts, including *BRAF* V600E mutations, although these mutations may be detected in premalignant lesions alongside *BRAF* V600K or *NRAS* mutations [29,30] and at low allele frequency in benign nevi [23].

For the high-CSD pathways (de novo pathways), MAPK activating mutations are weak, late, and/or absent during melanoma formation. In this pathway, a melanocyte on chronically sun-damaged skin accumulates mutations, sometimes forming a subclinical field of mutated melanocytes or premalignant intraepidermal melanocytic proliferation. Eventually, the cumulative burden of multiple weakly pro-oncogenic events results in melanoma formation. This pathway is thus responsible for the formation of high chronic sun damage melanomas such as lentigo maligna and desmoplastic melanoma that typically lack *BRAF* V600E mutation, but instead harbor mutations in *NRAS*, *NF1*, or non-V600E mutations in *BRAF* [9,21].

UV-independent tumorigenic pathways give rise to several rare melanoma subtypes. Acral lentiginous melanomas are often driven by oncogene amplifications such as *CCND1* or *CDK4* that are detectable in a field of background melanocytes [22]; alternatively, fusions or more classic oncogene point mutations might be present. UV-independent tumorigenic pathways for blue-nevus-like melanoma and Spitz melanomas are described in greater detail below.

In addition to alterations of specific genes, there are global differences associated with melanoma. Overall tumor mutation burden increases with progression to melanoma, although it has been challenging to define a distinct threshold that is highly sensitive and specific for distinguishing nevus from melanoma [29,31]. Melanoma is associated with increased genomic instability resulting in segmental chromosomal gains and losses that can allow for distinction from nevi [32,33]. Epigenetic shifts such as changes in global methylation and loss of hydroxy-5-methylcytosine can also be observed [34,35,36,37].

In summary, a broad array of potential founder oncogenic drivers contributes to a remarkable range of morphologic subtypes and variation in both benign and malignant melanocytic neoplasms. Additional morphologic complexity arises from secondary mutations in melanocytomas, and extreme heterogeneity in molecular changes related to malignant progression in melanoma. These factors explain (at least in part) substantial diagnostic challenges that can arise due to morphologic overlaps between benign and malignant neoplasms, as well as the occurrence of truly biologically intermediate neoplasms (i.e., those with a risk of adverse clinical course that is higher than a nevus but significantly lower than melanoma) (Figure 1B). These observations also support utility for molecular analyses in the diagnosis of challenging melanocytic lesions.

## 4. Immunohistochemistry Overview

While morphologic assessment is the most important diagnostic tool in the evaluation of melanocytic lesions [11], immunohistochemistry is the most frequently used adjunct when dealing with ambiguous or borderline lesions. As compared to molecular testing, immunohistochemistry has advantages that include shorter turnaround time, broader availability, ease of correlating results with specific morphologic populations, and often lower cost [13]. Immunohistochemical markers contribute in three broad ways to diagnosis (with markers potentially considered in more than one category): melanocytic markers, risk markers, and driver markers.

Melanocytic markers such as SOX10, S100, and MART-1 may be used to confirm melanocytic lineage or highlight lesional architecture [13]. Notably, MART1 expression may be lost in areas of some Spitz neoplasms, and in part or all of some melanomas.

Risk markers display differential staining patterns in nevi as compared to melanoma and include HMB45, p16, Ki67, and PReferentially expressed Antigen in MElanoma (PRAME), among others. In general, these markers are specific but have limited sensitivity; i.e., aberrancy favors melanoma, but lack of aberrancy is less informative.

HMB-45 (also a melanocytic marker) is most often used to demonstrate “maturation” of nevi as evidenced by staining superficially that diminishes with dermal descent. Melanoma classically lacks stratification, instead displaying aberrant patterns such as diffuse or heterogeneous staining, although stratification similar to nevi can be observed in some melanomas. Notably, diffuse HMB-45 expression is characteristic of pigment-synthesizing lesions, such as blue nevus or deep penetrating categories, and is not considered aberrant in this context [13,38,39].

As mentioned above, the tumor suppressor *CDKN2A* displays recurrent loss in melanoma, and thus immunohistochemistry demonstrating diffuse or zonal loss of expression of the *CDKN2A* gene product p16 raises concern for malignancy [13,19]. In nevi, p16 can display a mosaic (“checkerboard”) pattern with mixed positive and negative labeling. In contrast, true p16 loss requires complete lack of labeling in all melanocytes within an area of concern or throughout the entire lesion. This strict requirement can raise challenges to interpretation, with false positive aberrancy related to “low checkerboard” pattern due to a small fraction of positive melanocytes or failure to recognize aberrancy when p16 is lost in melanocytes but intermingled p16-positive histiocytes create the appearance of retention. As discussed below, fluorescence in situ hybridization (FISH) for *CDKN2A* loss may be a useful adjunct in such cases [13,40,41,42,43].

Ki-67 (especially when paired with MART-1 as a dual immunohistochemical stain) highlights the dermal melanocytic proliferation index. Higher proliferation indices are more often seen in melanoma [9]; proposed cutoffs vary, although >5% is often used. Similar to HMB45, proliferative labeling should also be relatively reduced in the deeper component of a nevus [13]. In definitive melanomas, an elevated proliferation index has been associated with an increased risk of metastasis [44]. Interpretive pitfalls include intermingled non-melanocytic cells such as inflammatory cells. Dual staining for Ki67 and MART1 improves (but does not eliminate) this challenge to interpretation. Spitz nevi, pregnancy-associated nevi, and special site nevi may show an elevated proliferation index that should not be confused with malignancy [13].

Immunohistochemistry for PRAME in the evaluation of melanocytic lesions has become widely used in recent years. PRAME is interpreted using a 0–4+ scale, as detailed in the seminal paper on this topic [45]. PRAME is positive (4+, >75% of melanocytic nuclei) in the majority of cutaneous melanomas and is negative or non-diffuse in the majority of benign nevi [45,46]. When compared with molecular diagnostic testing, PRAME immunohistochemistry displays generally high specificity and reasonable sensitivity in most studies, although there are mixed results regarding performance relative to molecular tests [47,48,49]. Some have proposed an alternative cutoff of 3+ (>50% positivity) to enhance sensitivity, potentially associated with lower specificity [50,51]. While there is often concordance between 4+ PRAME expression and a final diagnosis of melanoma in ambiguous lesions (such as with spitzoid or nevoid morphologies), agreement is not complete, and thus careful integration of multiple diagnostic parameters remains essential [52]. Considered broadly across tissue types and malignancies in general, PRAME expression is not synonymous with malignancy or even melanocytic lineage in isolation [46,53], so this result must be carefully considered with histomorphology, other immunohistochemical stains, and the molecular findings detailed herein.

Immunohistochemistry for driver events (discussed further below) includes detection of point mutations or overexpressed fusion products and can be used to help include or exclude specific morphologic variants. BRAF-V specifically detects the V600E mutant of BRAF without labeling V600K or non-V600 mutants. RAS Q61R highlights this activating point mutation in NRAS, HRAS, or KRAS. β-catenin patterning can indicate WNT activation status. Overexpressed fusion drivers include ALK, ROS1, and pan-TRK (NTRK1/2/3). In contrast to oncogene markers, null pattern (loss of expression) is the significant finding for tumor suppressor markers such as BAP1 and PRKAR1A. The indications for adding driver marker(s) to an immunohistochemistry panel are highly context-dependent, with details relevant to specific melanocytic classes below [10,11,12,13].

## 5. Classes of Melanocytic Neoplasm

### 5.1. Conventional/Ordinary (Acquired) Nevi

#### 5.1.1. Definition

Ordinary nevi are benign clonal proliferations of melanocytes that are comprised of nests containing at least three melanocytes [9]. Nevi can be junctional, compound, or dermal. Benign nevi are exceptionally common, representing the most common tumor found in humans [23].

#### 5.1.2. Molecular Pathogenesis

Ordinary nevi arise following an oncogenic founder mutation involving a single melanocyte, ref. [54] most often involving the MAPK signaling pathway including BRAF V600E or NRAS Q61R/L (Figure 1) [23,55]. A subset of nevi also demonstrates a higher mutational burden with enrichment of C->T mutations, compatible with an ultraviolet signature [23].

#### 5.1.3. Clinical Features

Benign nevi are typically circumscribed pigmented macules or papules, most frequently found on the trunk [9]. Typically, they demonstrate uniform pigmentation, size less than 6 mm, and reassuring dermatoscopic features [9,56]. Most acquired nevi arise during childhood and adolescence [57,58].

#### 5.1.4. Microscopic Features

**Figure 2:**
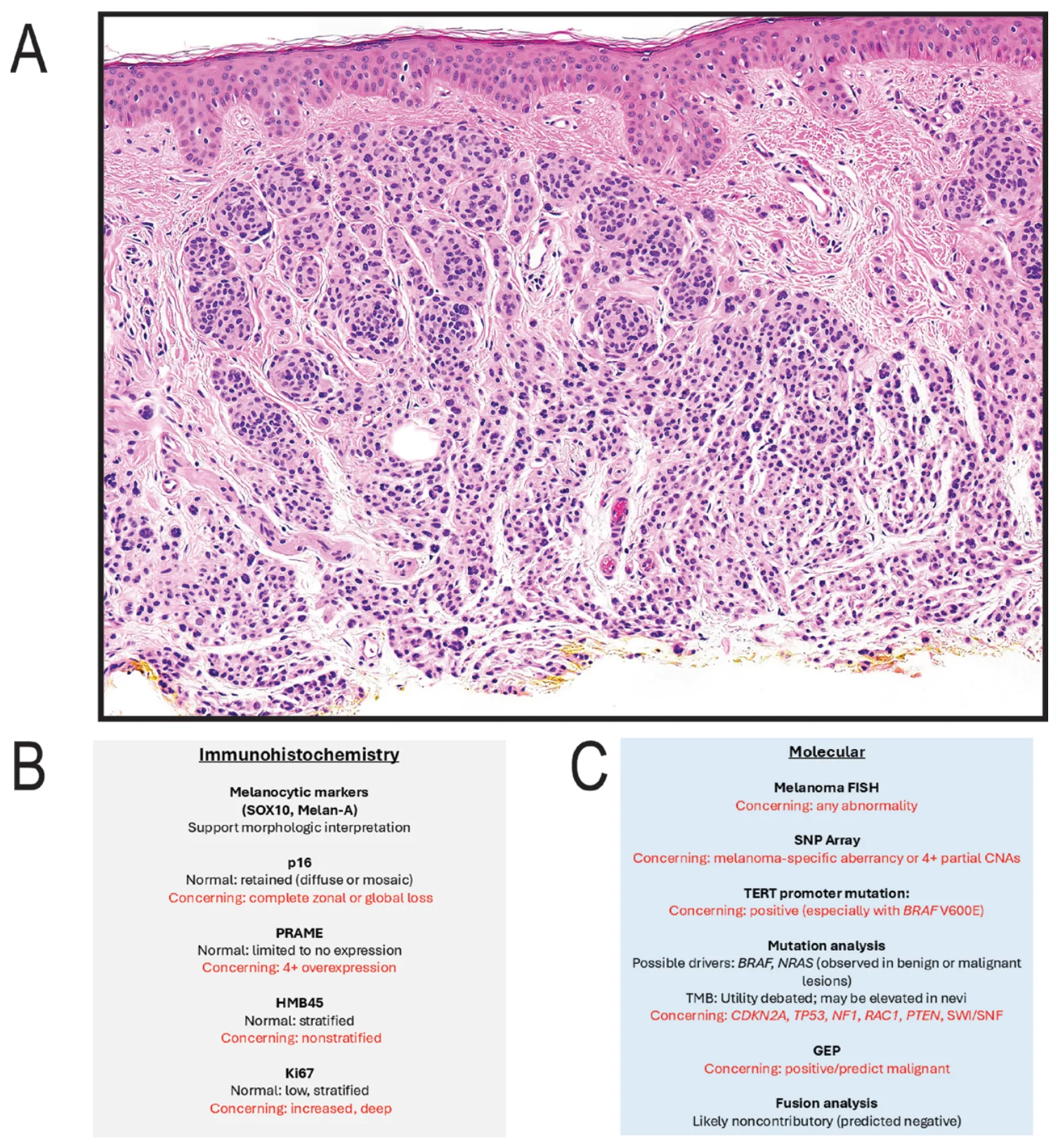
(**A**) Ordinary intradermal nevus demonstrating superficial nesting with maturation, bland cytomorphology, and no significant mitotic activity. Occasional multinucleated melanocytes are appreciated. H&E ×200. (**B**) Potential immunohistochemical (IHC) panel for conventional and dysplastic nevi. (**C**) Molecular diagnostic tests that may be informative for conventional and dysplastic nevi. As detailed in the text, results of ancillary IHC and molecular testing should be considered in the full context of findings for a given case.

By light microscopy, benign nevi demonstrate a circumscribed and symmetric silhouette with nests situated at the sides and tips of rete ridges. Lesions demonstrate banal cytomorphology, no significant mitotic activity, and mature with dermal descent in compound/dermal lesions (Figure 2). Superficial mitoses can be identified in specific clinical circumstances, such as childhood or pregnancy [59,60].

#### 5.1.5. Immunohistochemistry

Immunohistochemistry is not typically required for accurate diagnosis. However, a standard immunohistochemistry panel may be useful in challenging cases, such as when there is suspicion for nevoid melanoma (Figure 2B).

#### 5.1.6. Differential Diagnosis

The differential diagnosis includes dysplastic nevi and melanoma, both of which often display greater size, cellularity, degrees of asymmetry and architectural disorder, cytologic atypia, and mitotic activity [9]. Nevoid melanoma represents a rare but important pitfall with deceptively subtle atypia, accompanied by concerning findings including expansile dermal growth and increased mitotic activity. Most molecular diagnostic tests have been validated on common nevi and thus have the potential to provide diagnostic insight [11]. Ancillary testing that may be useful in challenging cases is summarized in Figure 2B,C.

### 5.2. Congenital Nevi

#### 5.2.1. Definition

Congenital nevi (CN) are benign melanocytic neoplasms that are present at birth or arise/become apparent within the first year of life.

#### 5.2.2. Molecular Pathogenesis

Congenital nevi arise due to in utero activating MAPK mutations, most frequently in *NRAS*. A minority of congenital nevi are associated with *BRAF* V600E or other less common mutations and fusions [9]. Histologically unremarkable congenital nevi tend to lack numerical chromosomal aberrations [61].

#### 5.2.3. Clinical Features

Congenital nevi are classified by predicted size in the adult, with <15 mm nevi being small, 15–200 mm intermediate, and >200 mm large/giant (with some reserving “giant” for nevi >400 mm) [9,62]. Potential complications include neurocutaneous melanosis or progression into melanoma.

#### 5.2.4. Microscopic Features

Congenital nevi extend to involve the deep dermis and often extend into the subcutaneous septa. There is tracking along dermal structures, including adnexal structures, nerves, and vessels. Collagen splaying is noted, with dermal nevomelanocytes forming cords between collagen bundles [9,63]. A maturation pattern is present, including decreasing cell size with descent into the dermis and dispersal at the base [63]. In childhood, the junctional component may display architectural disorganization, including pagetoid scatter [9,64].

#### 5.2.5. Immunohistochemistry

Immunohistochemical patterns are similar to those described above for acquired nevi (Figure 2B) [39,65].

#### 5.2.6. Differential Diagnosis

The diagnosis is usually straightforward. Acquired nevi may not be reliably distinguishable from small congenital nevi by histopathology alone, as the “congenital pattern” of dermal growth can also be observed in acquired nevi. Architectural disorganization of the intraepidermal component may raise consideration for a dysplastic nevus or even melanoma in situ.

There is risk of melanoma arising in congenital nevus, which is relatively low in smaller nevi but becomes increasingly significant in larger nevi [9,62]. The diagnostic challenges of melanoma arising in congenital nevus often relate to distinction from proliferative nodules, as discussed below. Ancillary testing that may be useful in challenging cases is similar to acquired nevi, as summarized in Figure 2B,C.

### 5.3. Proliferative Nodules

#### 5.3.1. Definition

Benign nodular proliferation of melanocytes arising in intermediate or large/giant congenital nevi.

#### 5.3.2. Molecular Pathogenesis

Proliferative nodules (PN) show identical driver mutations to the background CN [66]. Whole chromosomal gains and losses suggestive of mitotic spindle checkpoint defect can be observed in the proliferative nodule but not the background congenital nevus [67], suggesting that PNs might be classified in the category of melanocytoma [11]. Partial chromosomal changes can occur in PN but are rare [68]. Thus far, specific drivers responsible for initiating the formation of proliferative nodules have not been elucidated.

#### 5.3.3. Clinical Features

Clinically, proliferative nodules appear as a nodule or nodules arising in a congenital nevus. These can display rapid growth and (infrequently) ulceration [9,68].

#### 5.3.4. Microscopic Features

Scanning magnification demonstrates a dermal nodule with either gradual or abrupt transition from a background congenital nevus. Potential cytomorphologies include epithelioid, spindle cell, neurocristic, blue-nevus-like, spitzoid, or small round blue cell. The category of “atypical PNs” has been proposed for cases that display sharp demarcation, epidermal effacement, pleomorphism, and/or increased proliferation. Mitotic rate is generally low (<2 mitoses per square mm) but may be higher in small round blue cell or atypical PNs. High-grade atypia is absent [9,66,68].

#### 5.3.5. Immunohistochemistry

Negativity for PRAME has been described in most cases, although overexpression can occur [65,69]. There is retained expression of H3K27me3 [70,71]. Expression of KIT has been described in most proliferative nodules [72], although the molecular basis for this finding is unclear, as *KIT* amplification is not a recurrent alteration in PNs. Immunohistochemical patterns are otherwise generally similar to acquired nevi.

#### 5.3.6. Differential Diagnosis

The main distinction is from melanoma arising in a congenital nevus. The presence of multiple nodules favors a PN [68]. Melanoma often displays a higher mitotic count (5 or more per square mm) [68]. By immunohistochemistry, HMB45 can show homogeneous staining in both PN and melanoma [67]. Loss of p16 may be informative in adult-onset melanomas, but this finding has been reported to have low sensitivity for childhood melanomas arising in congenital nevi [67]. Melanoma can display segmental chromosomal gains and losses, including *RREB1* gains, unlike the whole chromosome changes typically observed in proliferative nodules [61,68]. Notably, a separate study of childhood melanomas arising in congenital nevi found low sensitivity for FISH [67]. *TERT*p mutations are not informative for distinguishing melanoma from proliferative nodules, as these are not found in either scenario [69,73].

Rarely, non-melanoma mesenchymal tumors such as embryonal rhabdomyosarcoma can also arise in congenital nevi [74,75] and must be distinguished from PNs.

### 5.4. Dysplastic Nevi

#### 5.4.1. Definition

While still benign, dysplastic nevi demonstrate atypical clinical and histopathologic features, including asymmetry, irregular pigmentation, architectural disorder, and cytologic atypia [9].

#### 5.4.2. Molecular Pathogenesis

Similar to common acquired nevi, dysplastic nevi are enriched with mutations involving the MAPK signaling pathway, including *BRAF* V600E or *NRAS* Q61R/L, as well as ultraviolet signature mutations on high cumulative sun damage sites [76]. Interestingly, specific additional genetic mechanisms contributing to the dysplastic phenotype have yet to be elucidated.

#### 5.4.3. Clinical Features

Dysplastic nevi are characteristically macular, with some having a central raised area imparting a “fried egg” appearance. The International Agency for Research on Cancer (IARC) consensus group has defined clinically atypical features to include: a non-well-defined border, size ≥ 5 mm, color variegation, an uneven peripheral contour, and the presence of erythema [77]. These features overlap with the clinical appearance of melanoma. Patients with high-grade dysplastic nevi are at elevated risk for the development of melanoma, which increases with the size and number of such nevi identified [78].

#### 5.4.4. Microscopic Features

**Figure 3:**
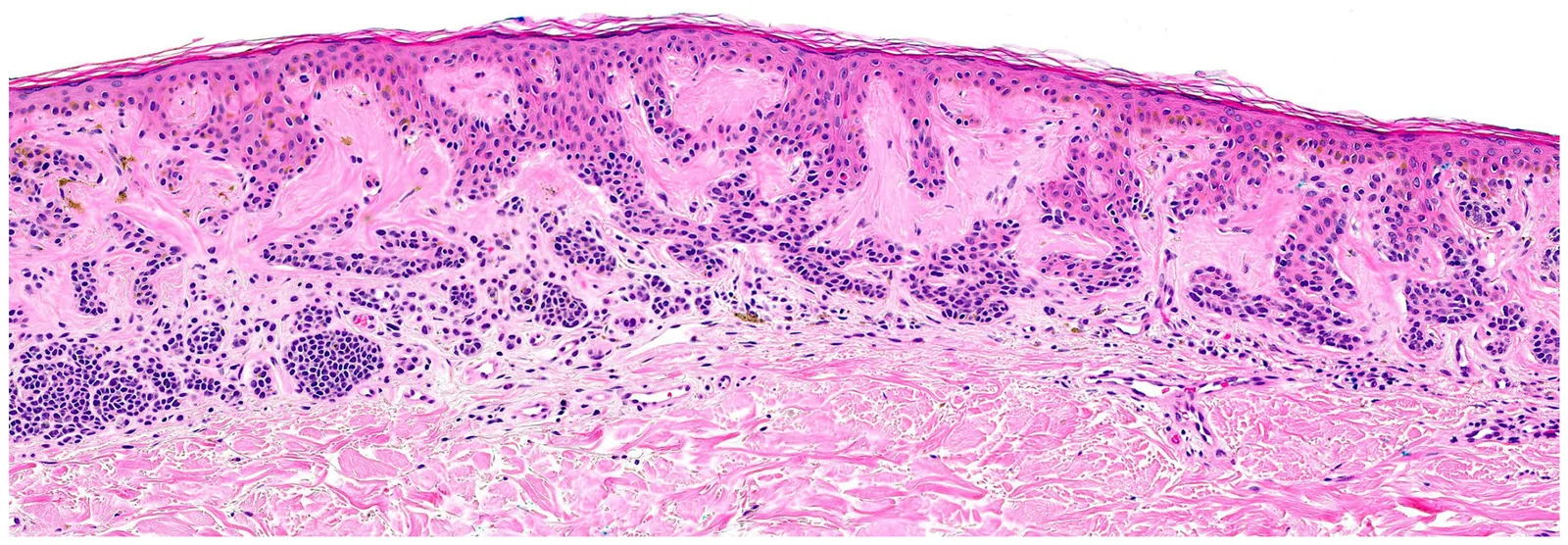
Compound dysplastic nevus demonstrating cytologic atypia bridging between adjacent rete ridges and shoulder architecture. H&E ×200.

Dysplastic nevi are often characterized by asymmetric growth with a junctional shoulder (extension of the junctional component beyond the lateral confines of the dermal component), bridging between adjacent rete ridges, and lentiginous growth with minor involvement of suprapapillary plates (Figure 3). Concentric or lamellar fibroplasia is often seen in the papillary dermis. Cytologic atypia is often a defining feature, ranging from mild to severe. Mitotic figures are inconspicuous [9].

#### 5.4.5. Immunohistochemistry

While not always required for the diagnosis of dysplastic nevi, immunohistochemistry can be useful in high-grade or borderline cases. A standard immunohistochemical panel can be informative (Figure 2B).

#### 5.4.6. Differential Diagnosis

The differential diagnosis includes ordinary nevi and melanoma. Melanoma often displays a greater degree of cytologic atypia, architectural disorder, cellularity, and mitotic activity. Immunophenotypic aberrancy is also more frequently encountered in melanoma [79]. Ancillary testing that may be useful in challenging cases is summarized in Figure 2B,C.

### 5.5. Blue Nevi and PKC-Fused Melanocytic Neoplasms

#### 5.5.1. Definition

Blue nevi are intradermal melanocytic neoplasms that are often pigmented and may be variably cellular, comprised of dendritic to spindled/ovoid melanocytes [9].

#### 5.5.2. Molecular Pathogenesis

Blue nevi are molecularly distinguished by the presence of constitutively activating driver mutations involving genes in the Gα_q/11_ signaling pathway (Figure 1), typically lacking the canonical genetic alterations of *BRAF*, *NRAS*, or *NF1* occurring in conventional melanocytic neoplasms [80,81]. The most mutated gene is *GNAQ*, with the initial study showing that 83% of blue nevi harbor an activating *GNAQ* Q209 mutation [80]. Glutamine 209 is within the Ras-like domain of *GNAQ*, and alteration of this site impairs GTPase activity, preventing GTP hydrolysis and resulting in constitutive activation [80]. Larger later studies revealed the presence of a *GNAQ* codon 209 mutation in a more modest 53–59% of cases, with alternative *GNA11* mutations found in 15–16% [81,82,83,84]. In one cohort, *GNAQ* exon 5 mutations were more frequent in cellular blue nevi than in ordinary blue nevi, occurring in 76% and 37%, respectively [82]. Other genes in the pathway such as *CYSLTR2* and *PLCB4* may also rarely be mutated and function as drivers of cellular proliferation [85]. Interestingly, *CYSLTR2* mutant blue nevi show significant morphologic overlap with pigmented epithelioid melanocytomas (PEMs; discussed further below), showing a heavily pigmented and exophytic phenotype [86]. Blue nevi involving lymph nodes, which may mimic metastatic melanoma, show a similar molecular profile to those occurring in the skin and have an indolent clinical course [87].

Beyond activating point mutations, gene fusions involving protein kinase C (PKC) isoforms, including *PRKCA* (most common), *PRKCB*, and *PRKCG*, as well as in *GRM1*, which encodes a G-protein coupled receptor expressed in the central nervous system, have been identified in blue nevi and related melanomas [88,89,90]. Although PKC-rearranged neoplasms were originally thought to represent a subset of pigmented epithelioid melanocytoma (PEM; discussed below) [91], PKC translocations in fact cause activation of the Gα_q/11_ pathway rather than signaling through the Gα_s_/cAMP/PKA axis (a feature of PRKAR1A-inactivated PEMs) [88]. Transcriptomic findings support placement of PKC-fused melanocytic neoplasms within the blue family [88].

Nevi of Ota, choroidal nevi, and uveal melanomas molecularly resemble blue nevi with respect to driver mutations. Of the four examples of *GRM1*-fused blue melanocytic neoplasms reported to date, two were plaque-like and one was classified morphologically as nevus of Ota [88].

So-called “malignant blue nevi” or blue-nevus-like melanomas (BNLMs), which have an anatomic predilection for the scalp and often arise in the context of a pre-existing common or cellular blue nevus, often have secondary mutations in *BAP1*, *SF3B1*, or *EIF1AX*, which are not seen in benign cellular blue nevi (Figure 1) [83,84,92]. Mutations in other genes related to genomic instability, such as *KMT2C*, *FANCD2*, *ATR*, and others, may also be found, as well as mutations involving the PI3K pathway [93]. Interestingly, approximately 70% of BNLMs have mutations in *GNA11*, as opposed to only approximately 10% of cellular blue nevi, suggesting a higher malignant potential than activating *GNAQ* variants [83,92]. In contrast to conventional melanoma, *TERT*p mutations are infrequent in BNLM, which also tend to have a low tumor mutational burden, similar to uveal melanoma [92]. Clinically and histopathologically, BNLMs tend to have a significant Breslow depth with a high rate of microscopic satellitosis, sentinel lymph node involvement, and/or distant metastasis at presentation [92]. Although BNLMs tend to present at an advanced stage, prognosis may not be significantly different from conventional melanomas displaying similar clinicopathologic features [14,94].

BNLMs also show significant differences from their benign counterparts by copy number analysis. While cellular blue nevi tend to lack copy number alterations (CNAs), BNLMs have detectable copy number aberrations in most cases by single-nucleotide polymorphism (SNP) microarray or array-based comparative genomic hybridization (aCGH) [83,89,95].

**Figure 4:**
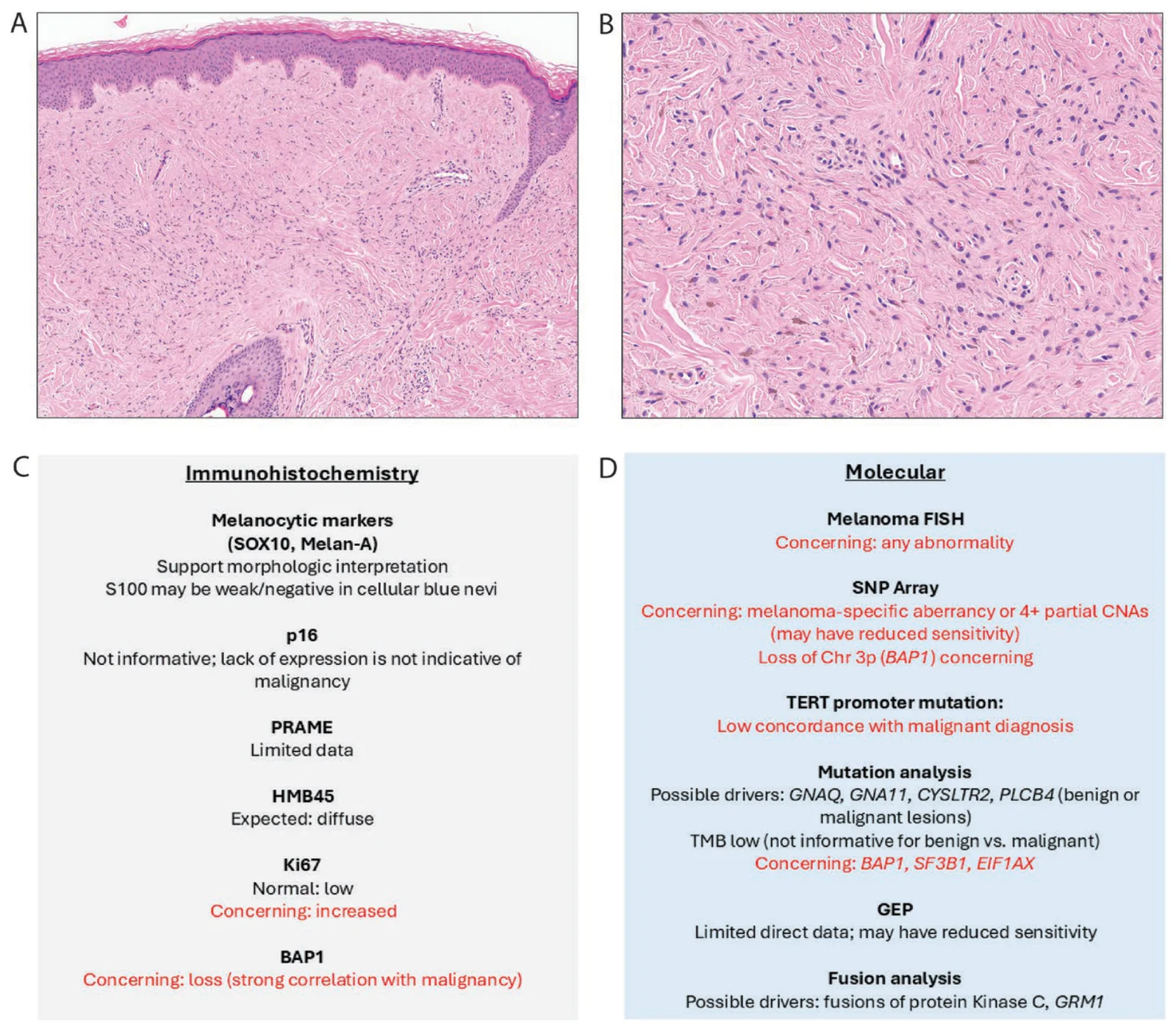
Common blue nevus. (**A**) Intermediate magnification image revealing an intradermal proliferation of melanocytes within a collagenous background. (**B**) High magnification reveals spindled to dendritic melanocytes containing hyperchromatic nuclei and lacking cytologic atypia. Scattered melanophages are seen in the background. H&E. Magnification: (**A**) ×16 (**B**) ×400. (**C**) Potential immunohistochemical (IHC) panel for blue nevi (including those with PKC gene fusion). (**D**) Molecular diagnostic tests that may be informative for blue nevi (including those with PKC gene fusion). As detailed in the text, results of ancillary IHC and molecular testing should be considered in the full context of findings for a given case.

Copy number complexity appears to increase across the biologic spectrum of blue nevus family tumors. Atypical cellular blue nevi, which are regarded as melanocytomas in the current WHO Classification of Tumours by morphological findings rather than a known second genetic “hit” [9,14], showed a single CNA in 25% of cases, with no cases displaying multiple CNAs [95]. In contrast, most BNLMs had multiple abnormalities, including recurrent whole-arm gains of 1q, 4p, 6p, and 8q and losses of 1p and 4q [83,95]. BAP1 expression loss may be associated with copy number loss on 3p [83]. The absence of these changes from histologically benign precursor areas supports their acquisition during malignant progression [95]. Notably, recurrent mutations specific to malignancy in the context of blue nevi (such as *BAP1* and *SF3B1* R625 mutations) are not detected by SNP array [83,84,92,96]. Ancillary testing that may be useful in challenging cases is summarized in Figure 4B,C.

#### 5.5.3. Clinical Features

Blue nevi typically present as blue–gray to black macules or papules less than 1 cm in size. Uncommonly, blue nevi can be amelanotic. Rare subtypes include agminated, eruptive, linear, targetoid, and plaque-type blue nevi [9].

#### 5.5.4. Microscopic Features

Blue nevi are most often intradermal and comprised of melanocytes with dendritic, spindled, or ovoid nuclear contours. Stromal sclerosis and numerous associated melanophages are classic [9] (Figure 4).

**Figure 5:**
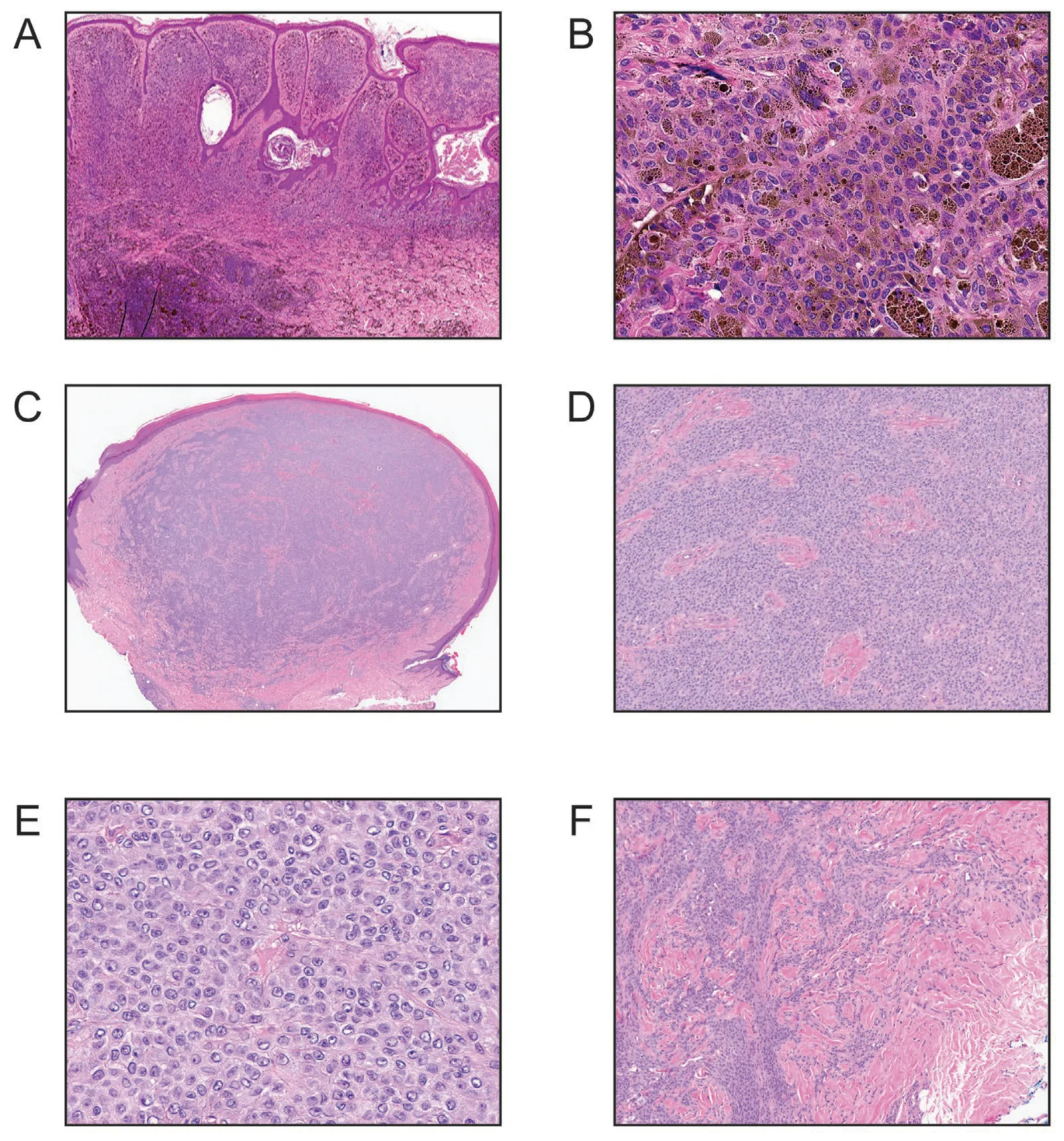
PKC-fused melanocytic neoplasm (atypical cellular blue nevus/low-grade blue melanocytoma). (**A**,**B**) Example case with heavy pigmentation. (**A**) Hyperpigmentation with exophytic component on scanning. (**B**) Deeply pigmented melanocytes. (**C**–**F**) Example hypopigmented case. (**C**) Scanning magnification reveals a hypopigmented intradermal melanocytic proliferation with an exophytic growth pattern. (**D**) The center of the lesion shows large coalescing nests of melanocytes within a sclerotic background. (**E**) High magnification reveals a monomorphic population of epithelioid cells with small nucleoli but without high-grade cytologic atypia. The mitotic rate is low (1/mm^2^), with a Ki67 proliferation index of 1%. (**F**) Areas at the periphery show a biphasic morphology with spindle cells intermixed with epithelioid forms. H&E. Magnification: (**A**) ×16, (**B**) ×400, (**C**) ×17 (**D**) ×100 (**E**) ×400 (**F**) ×100).

Compared with PRKAR1A-inactivated PEMs, PKC-fused melanocytic tumors tend to present earlier in life and more commonly form cohesive aggregates/nests of melanocytes, often with a biphasic appearance resembling so-called “combined blue nevi”, a collagenous stroma, and smooth muscle hyperplasia [88,90,97] (Figure 5).

#### 5.5.5. Immunohistochemistry

Melanocytes in blue nevi are characteristically diffusely positive for HMB45 in addition to other markers of melanocytic lineage (Figure 4C) [9]. There is debate regarding the potential for benign blue nevi to lack p16 expression [98,99,100,101]; in practice, it seems likely that the “low checkerboard” pattern of retained expression may be challenging to reliably distinguish from absent p16 expression, especially in the background of intermingled p16-positive histiocytes. S100 can be weak or negative in cellular blue nevi; however, other melanocytic markers are robustly expressed [98].

#### 5.5.6. Differential Diagnosis

The spindled cytomorphology and sclerotic stroma of blue nevi may raise consideration for desmoplastic melanoma in some cases; however, blue nevi should have no significant cytologic atypia and are positive for HMB45 and Melan-A [9]. Cellular blue nevi are densely cellular lesions with deep pushing borders but still lack definitive features of malignancy [9].

Notably, mutations involving the Gα_q/11_ pathway are not found in conventional or Spitz melanocytic neoplasms (Figure 1A) [80,102].

BNLMs show expansile asymmetric growth, a greater degree of cytologic atypia, mitotic activity, immunophenotypic aberrancy, and additional molecular aberrations detailed above [9]. Loss of BAP1 protein expression by immunohistochemistry is concerning for BNLM relative to benign blue nevi, although not completely sensitive or specific [10,83,95,103].

Molecular testing considerations in challenging cases may include FISH or SNP array. Mutation analysis and fusion testing can confirm the presence of blue nevus drivers, and larger mutation panels may highlight mutations associated with malignant progression. Roles for *TERT*p mutation testing and gene expression profiling are less clear.

An additional consideration is the potential for blue nevi or BNLM to be confused with dermal metastatic melanoma. Blue-nevus-like morphology metastatic melanoma can pose a diagnostic challenge and can be morphologically indistinguishable from blue nevi. Care should be given in lesions with blue-nevus-like cytomorphology in close proximity to a previously diagnosed melanoma, with consideration given to additional immunohistochemical and molecular studies for further evaluation [12]. Approaches to this distinction are detailed in the “Clonality testing to distinguish metastases from new primary” section below.

### 5.6. Spitz Nevi and Melanocytomas

#### 5.6.1. Definition

Lesions now classified as Spitz neoplasms were first described in 1948. Ref. [104] Spitz neoplasms range from benign (Spitz nevi) to borderline or intermediate morphology (Spitz melanocytoma), to malignant (Spitz melanoma). Such lesions are most often comprised of enlarged epithelioid to spindled melanocytes [9].

#### 5.6.2. Molecular Pathogenesis

**Figure 6:**
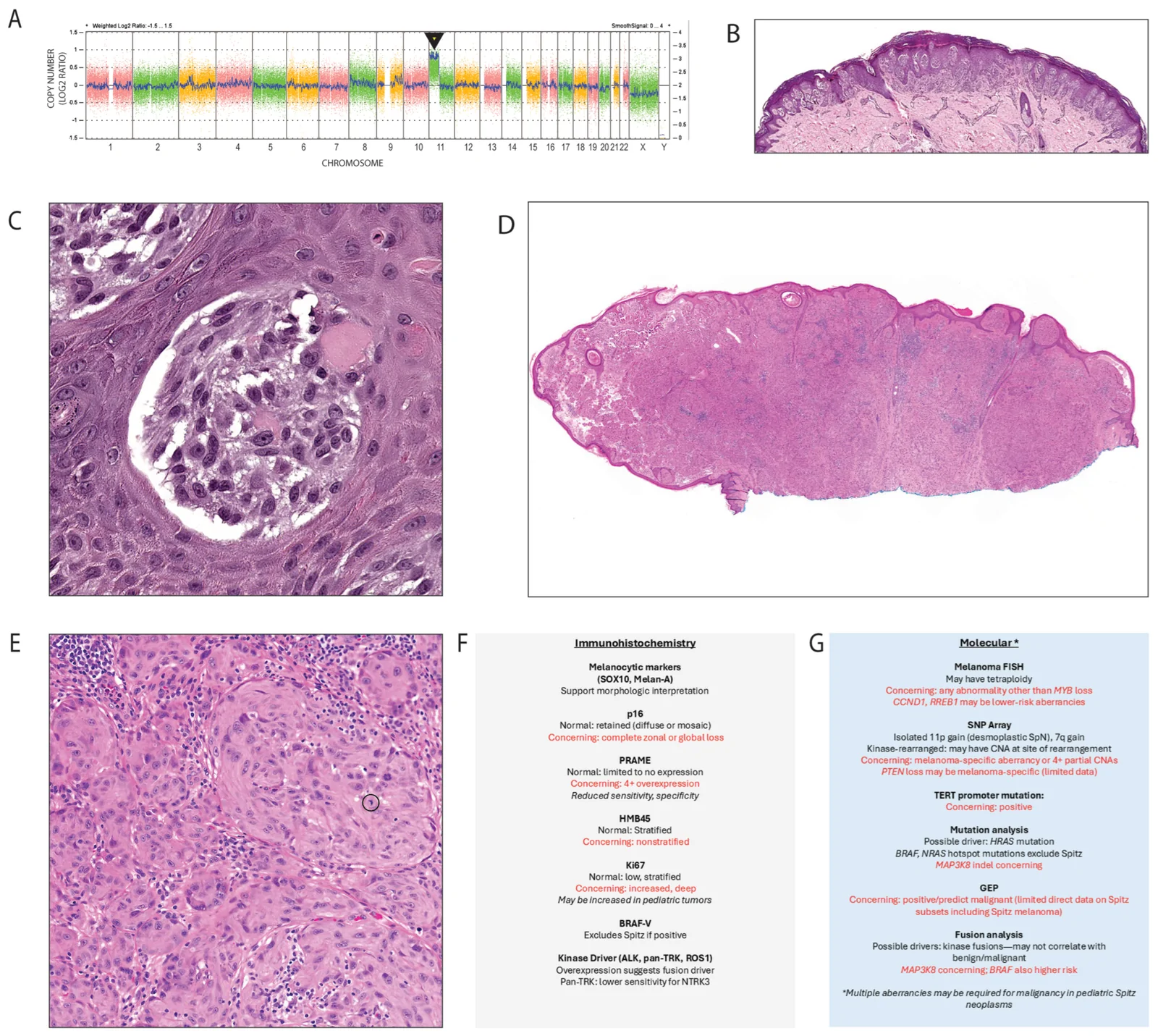
Spitz nevus and melanocytoma. (**A**) SNP array demonstrating isolated chromosome 11p gain (yellow arrowhead) classic for a subset of Spitz nevi/melanocytomas. Chromosomes are differentially colored for visual clarity. (**B**) Spitz nevus, demonstrating a circumscribed and symmetrical melanocytic lesion with associated epidermal hyperplasia. (**C**) Spitz nevus nest with clefting and associated Kamino body. Spitz melanocytes demonstrate abundant cytoplasm and a small nucleolus. (**D**) Spitz melanocytoma: dome-shaped compound melanocytic lesion with expansile growth. (**E**) Spitz melanocytoma: densely cellular nests with Spitz cytomorphology and identifiable mitotic figures at the mid-portion of the lesion (circled). (**B**–**E**) H&E. Magnification: (**B**) ×15, (**C**) ×400, (**D**) ×27, (**E**) ×200. (**F**) Potential immunohistochemical (IHC) panel for Spitz neoplasms. (**G**) Molecular diagnostic tests that may be informative for Spitz neoplasms. As detailed in the text, results of ancillary IHC and molecular testing should be considered in the full context of findings for a given case.

True Spitz neoplasms harbor defining genetic alterations that set them apart from other lesions demonstrating “spitzoid” cytomorphology. Spitz-defining mutations include *HRAS* mutations (and/or gain of 11p), activating fusions of receptor tyrosine kinases (*ALK, FGFR, ROS1, NTRK1, NTRK2, NTRK3, MET, MERTK*, and *RET)* and activating fusions of intracellular serine/threonine kinases (*BRAF, RAF1*, or *MAP3K8)* (Figure 1A and Figure 6A) [10,12,14,105,106,107,108,109,110,111,112,113,114].

Apart from an initiating mutation or fusion supporting Spitz lineage, Spitz nevi often lack additional abnormalities such as *TERT*p mutations, homozygous loss of *CDKN2A*, or significant CNAs (apart from gain of 11p, gain of 7q, and tetraploidy) [9,115,116,117]. Spitz melanocytomas can display similar fusions to Spitz nevi and represent an exception to the concept that melanocytomas harbor a second oncogenic driver [14].

Some studies suggest that by array CGH, Spitz melanocytomas have profiles distinct from Spitz nevi and melanoma [118]. The initially proposed four-probe melanoma FISH panel including *RREB1, MYB, CCND1*, and *CEP6* had limited sensitivity or specificity in the evaluation of borderline Spitz neoplasms [118,119]; yet, the utility increases with the addition of probes targeting *MYC* and *CDKN2A* [119,120]. *TERT*p mutations and *CDKN2A* homozygous deletion have been associated with more aggressive clinical behavior, although, as isolated findings, these may not be sufficient to predict aggressive behavior in childhood tumors [121,122].

Specific driver fusions have also been proposed to correlate with benign or malignant status. Spitz melanomas are enriched for *MAP3K8* fusions or truncating mutations (activating the kinase via truncation, resulting in loss of the regulatory domain) and to a lesser extent *MET* and *BRAF* fusions [10,107,123,124]. Notably, tumors harboring these drivers are not universally malignant [107,124,125,126]. In contrast, most Spitz lesions with *ALK, NTRK1, NTRK3, RET*, and *ROS1* fusions are Spitz nevi or melanocytomas [124]. However, in some cases, Spitz melanomas can involve kinases more characteristic of Spitz nevi/melanocytomas [124]; thus, the fusion driver by itself should not be considered sufficient evidence to exclude or confirm a diagnosis of melanoma.

A recent comprehensive study of seven childhood Spitz melanomas with aggressive course found that these tumors were characterized by a driver fusion (*MAP3K8, ALK*, or *BRAF*) combined with additional hits, including *MYC* aberrations; *CDKN2A* deletion combined with *PTEN* deletion or *TERT*p mutation; or complex chromosomal copy number changes [122]. Thus, comprehensive molecular analysis may be optimal to achieve the greatest diagnostic precision in such cases.

#### 5.6.3. Clinical Features

Spitz lesions are often papules or nodules and have a predilection for the face and extremities of children but can occur at any age [127,128,129,130,131]. Lesions tend to display rapid growth over months and then stabilization [9]. Spitz nevi are often solitary, circumscribed, symmetric, and measure < 6 mm in size [129].

Distinction between a Spitz nevus and Spitz melanocytoma is challenging and nonspecific on clinical grounds alone; however, Spitz melanocytomas may be associated with irregularity in pigmentation, increased size, or ulceration [132,133]. Like blue melanocytomas, Spitz melanocytomas are defined morphologically rather than by the presence of a known second genetic “hit” [9]. While clinically and histologically atypical, Spitz melanocytomas often follow a benign/indolent clinical course. Sentinel lymph node biopsy is generally contraindicated in these cases, as approximately 40% are positive without accurate prediction of aggressive behavior [134,135,136,137].

#### 5.6.4. Microscopic Features

A “classic” Spitz nevus demonstrates symmetric, circumscribed, and wedge-shaped growth with associated epidermal hyperplasia (Figure 6B). The junctional component is predominantly nested, with vertically arranged nests accompanied by characteristic clefts between the nests and adjacent epidermis. Upward scatter is often confined to the center of the lesion, and may include nests as well as individual melanocytes [138]. Scattered eosinophilic globules termed Kamino bodies can often be observed [139]. Cytologically, Spitz melanocytes demonstrate enlarged and pleomorphic nuclei with prominent nucleoli and abundant cytoplasm. Spindled and smaller epithelioid cytomorphology can also be seen. Like conventional nevi, Spitz nevi show at least some evidence of maturation with dermal descent, although deeper melanocytes retain Spitz cytomorphology. Mitotic figures can be appreciated but are often <2 mitoses/mm^2^ and should ideally be confined to the superficial aspect of the lesion [129,131,140] (Figure 6C,D).

Many variants of Spitz nevi have been described. In recent years, correlation of morphology with molecular driver has become more well-defined. For instance, desmoplastic Spitz nevi (defined by sclerotic stroma) are most often characterized by *HRAS* activating mutations, often accompanied by gains in chromosome 11p involving the *HRAS* gene [105,141,142]. The pigmented spindle cell nevus (of Reed) is a predominantly junctional lesion with high cellularity and dense melanin pigment with enrichment for *NTRK3* fusions [143,144]. Spitz nevi with *ALK* fusions can demonstrate intersecting short fascicles of spindle cells [145,146]. Lesions with *NTRK1* rearrangements have been reported to display filagree-like rete ridges, rosettes, and nests with exaggerated maturation [147]. *MET* fusions tend to result in lesions with spindled cytology and plexiform dermal growth [148]. Lesions with *RET* fusions more often display confluent nesting with superficial discohesion, prominent Kamino bodies, multinucleation, and increased vascularity [149]. Additional morphologic variants include the pagetoid Spitz nevus, which shows prominent upward scatter, raising a risk for misdiagnosis as melanoma in situ; the molecular drivers for this Spitz subtype are less well-characterized, although *NTRK3* fusion was described in one case [150].

In contrast to Spitz nevi, Spitz melanocytomas display an expansile and pushing silhouette at scanning magnification. In addition, there are often other features present not typically seen in Spitz nevi, such as extensive pagetoid scatter, thinning of the epidermis, ulceration, lack of maturation, and increased dermal mitotic activity, particularly at the leading edge of the tumor [9] (Figure 6D,E).

#### 5.6.5. Immunohistochemistry

A possible immunohistochemical panel for Spitz neoplasms is summarized in Figure 6F. p16 is often retained, although loss by immunohistochemistry does not always equate to homozygous *CDKN2A* deletion or malignancy [10,42,43,151]. PRAME has relatively lower specificity, with the potential for overexpression in Spitz nevi [45,152]. If BRAF-V immunohistochemistry is positive, the lesion is best classified as spitzoid rather than truly Spitz. Markers such as ALK, pan-TRK, ROS1, and MET are becoming more widely used to demonstrate overexpression suggestive of a fusion [10,129]. Notably, pan-TRK has limited sensitivity for detecting NTRK3 overexpression and often shows nonspecific reactivity [153].

#### 5.6.6. Differential Diagnosis

The most important entity to exclude in the evaluation of Spitz and spitzoid neoplasms is melanoma. Features more commonly encountered in melanoma include size >10 mm, ulceration, >2 mitoses/mm^2^, patient age > 40 years, subcutaneous extension, diffuse pagetoid scatter, angiolymphatic invasion, increased cellularity, and, while subjective, marked cytologic atypia [127,133,154,155].

There is historically poor interobserver variability on the classification of Spitz neoplasia [155]. However, there have been recent studies indicating improvement in interobserver agreement, in particular defining lesions within the Spitz lineage versus conventional melanoma, with the incorporation of next-generation sequencing (NGS) data [156,157]. The most useful information gleaned from NGS is the detection of *BRAF* and *NRAS* mutations, which exclude Spitz lineage [156,157]. Additionally, *BRAF* and *RAF1* fusions are not specific to Spitz neoplasms, so careful correlation with morphologic features and additional molecular studies (such as SNP array and *TERT*p mutation status) is recommended to exclude non-Spitz melanoma [158,159,160]. However, once molecularly established as a Spitz neoplasm, no single histopathologic or molecular feature is diagnostic of Spitz melanoma. Apart from long-term follow-up demonstrating metastasis or death from disease, there is no consensus on what distinguishes melanoma from high-grade atypical Spitz tumors, underscoring the need for molecular tools to improve risk stratification in challenging cases. For example, cases with expansile growth, deep mitotic figures, elevated proliferative index, loss of p16 by immunohistochemistry, and/or PRAME positivity may benefit from additional molecular evaluation. Large numbers of CNAs, especially involving genes frequently encountered in melanoma, as well as *TERT*p mutation, should raise significant suspicion for melanoma [121]. Ultimately, incorporation of molecular results with overall morphologic, immunophenotypic, and clinical (such as patient age) features is essential.

Importantly, a category of melanocytic neoplasms with spitzoid cytomorphology and *BRAF* V600E mutations (termed *BRAF*-mutated and morphologically spitzoid, BAMS) has been described [161]. Such lesions are challenging to distinguish from true Spitz lesions, even for expert dermatopathologists [161]. Histopathologically, BAMS show fewer Kamino bodies and increased pigmentation, but otherwise similar histopathologic features as Spitz lesions with conventional initiating driver mutations or gene fusions [162]. Next-generation sequencing reveals frequent “second-hit” mutations in the *KMT* family, intermediate in number between nevi and melanoma [162]. The current understanding of BAMS best categorizes them as melanocytomas in the setting of reassuring immunohistochemistry, lack of *TERT*-p mutations or abnormalities by FISH, and absence of overtly malignant morphologic features [162].

Considering these observations, molecular testing for challenging Spitz tumors might include multiple modalities, including fusion analysis (for kinase driver type), oncogene mutation testing (for *BRAF* or *NRAS* drivers that would exclude Spitz), SNP array or melanoma FISH, and *TERT*p mutation testing (Figure 6G). Gene expression profiling has been tested on Spitz nevi, although data on Spitz melanoma are limited; thus, the sensitivity for this modality remains unclear.

### 5.7. Pigmented Epithelioid Melanocytomas

#### 5.7.1. Definition

Pigmented epithelioid melanocytomas (PEMs) are defined as pigmented epithelioid tumors with an initial driver mutation from either the conventional (most commonly *BRAF*), blue, or Spitz pathways and secondary activation of protein kinase A (PKA), frequently associated with inactivating mutations in the gene encoding the PKA regulatory subunit PRKAR1A [163,164]. Historically, these tumors have been referred to under several names such as epithelioid blue nevus and “animal-type” melanoma [165].

#### 5.7.2. Molecular Pathogenesis

PEMs are proposed to be associated with an initial driver mutation, followed by an activation event of PKA resulting in increased signaling through the Gα_s_ pathway (Figure 1). The most frequently encountered founder mutation is *BRAF* V600E, although a broad spectrum of founder drivers has been described [144]. Although PKA activation occurs most characteristically via inactivation of PRKAR1A [9], the current WHO Classification of Skin Tumors does not strictly require PRKAR1A inactivation, allowing for the possibility of additional as-yet-undefined PKA activating events to also culminate in this distinctive morphology [9].

PEM-like melanomas (PRKRAR1A-inactivated melanomas) may represent malignant progression of PEM or secondary PRKAR1A inactivation in a background of conventional melanoma. Such melanomas often contain *TERT*p mutations as well as numerous CNAs, changes not seen in morphologically low-grade PEMs [163]. *TP53* and *RB1* mutations have also been reported in PEM-like melanomas [143]. Borderline cases, termed “high-grade” or “atypical” PEMs, have also been described [163].

#### 5.7.3. Clinical Features

PEMs may occur sporadically as well as in the context of the Carney complex, characterized by germline mutations, primarily in *PRKAR1A* [166]. Other manifestations of the Carney complex include superficial angiomyxomas, cardiac myxomas, primary pigmented nodular adrenal disease, melanotic schwannomas, widespread lentigines, and café au lait spots [166].

Like other melanocytomas, the vast majority of PEMs behave in an indolent fashion despite regional lymph node involvement in a high proportion of cases (nearly 50% in small series) [165,167].

#### 5.7.4. Microscopic Features

**Figure 7:**
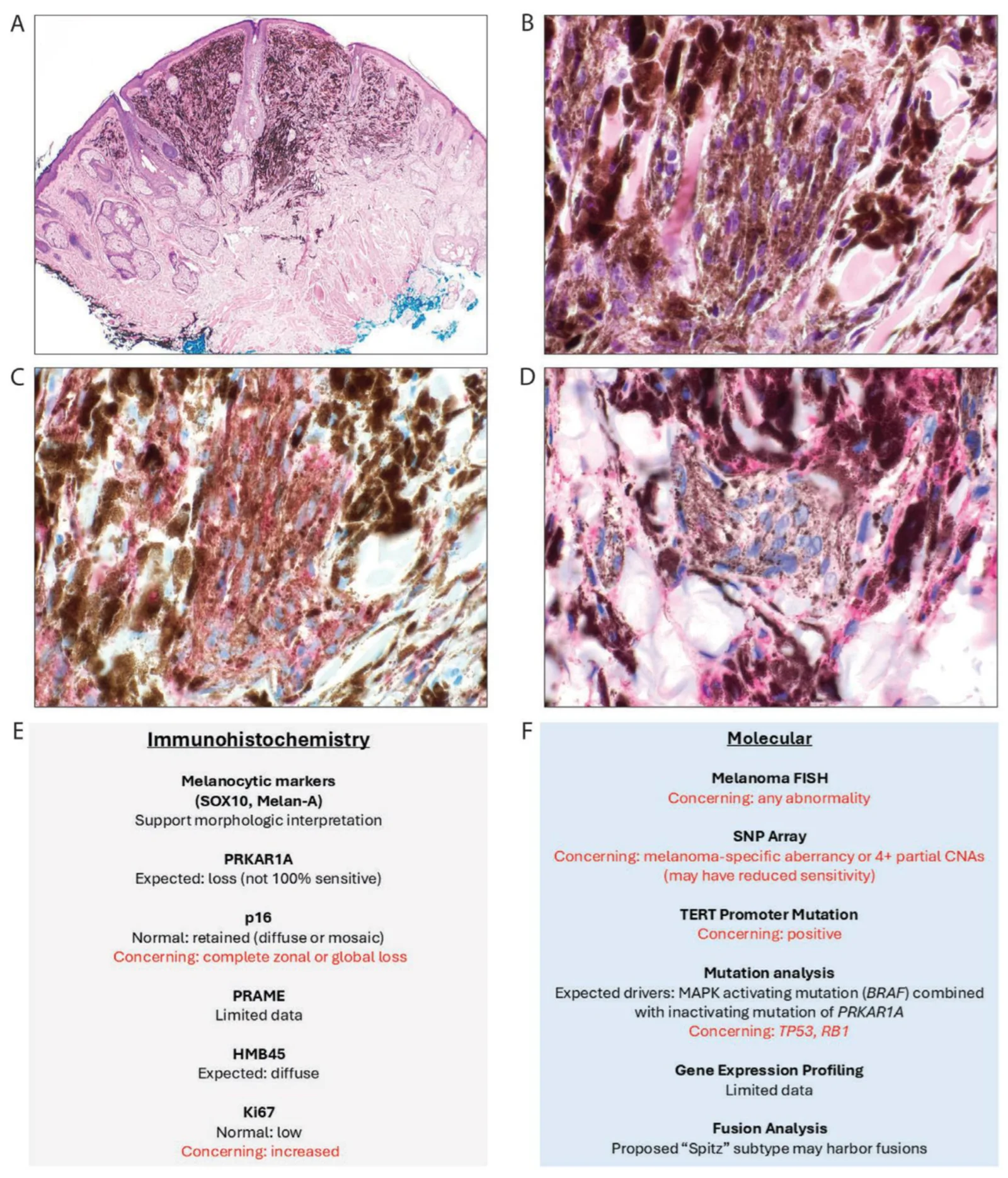
Pigmented epithelioid melanocytoma. (**A**) Scanning magnification image revealing an exophytic, heavily pigmented intradermal melanocytic lesion. (**B**) High magnification reveals small nests of epithelioid melanocytes with abundant cytoplasmic melanin pigment and enlarged oval nuclei with small nucleoli. Numerous melanophages are present in the background dermis. (**C**) The melanocytes are diffusely positive for HMB-45 by immunohistochemistry. (**D**) Immunohistochemistry for PRKAR1A reveals loss of expression in the tumor cells. (**A**,**B**) H&E. (**C**,**D**) Red chromogen with hematoxylin counterstain. Magnification: (**A**) ×40 (**B**–**D**) ×600. (**E**) Potential immunohistochemical (IHC) panel for pigmented epithelioid melanocytoma. (**F**) Molecular diagnostic tests that may be informative for pigmented epithelioid melanocytoma. As detailed in the text, results of ancillary IHC and molecular testing should be considered in the full context of findings for a given case.

PEMs show characteristic clinical and histopathologic features and are bulbous or wedge-shaped, predominantly intradermal, and comprised of large, epithelioid melanocytes with enlarged, pleomorphic nuclei containing vesicular chromatin and prominent nucleoli [9,14]. The melanocytes often contain pigmented cytoplasm and are associated with abundant intermixed melanophages. Dendritic and spindled melanocytes may also be present (Figure 7). A background precursor nevus may be identified.

#### 5.7.5. Immunohistochemistry

A possible immunohistochemistry panel is summarized in Figure 7E. Loss of PRKAR1A expression by immunohistochemistry is a useful surrogate for genetic testing [10,168] but is not completely sensitive for PEMs [14].

#### 5.7.6. Differential Diagnosis

The three different subclasses of PEM may show some morphologic overlap with blue nevi, conventional nevi, or Spitz nevi. As previously discussed, PKC-rearranged cases were originally considered to be PEMs but are now classified within the blue nevus family [88].

The most important differential diagnosis involves distinction of PEM from PRKAR1A-inactivated/PEM-like melanoma. Molecular testing in challenging cases (summarized in Figure 7F) might include mutation profiling to confirm the PEM diagnosis and evaluate for concerning mutations such as *TP53* or *TERT*p. SNP array can detect copy number abnormalities but may have reduced sensitivity.

### 5.8. WNT-Activated Deep Penetrating/Plexiform Melanocytoma (Naevus)

#### 5.8.1. Definition

Deep penetrating nevi (DPN) are now classified as “melanocytomas” in the current *WHO Classification of Tumours*, now termed “Wnt-activated deep penetrating/plexiform melanocytoma (naevus)” (WNT-activated DPMNs) [9,14].

#### 5.8.2. Molecular Pathogenesis

In the case of DPN, the initial mutation is most commonly *BRAF* V600E (less frequently *MAP2K1* in-frame deletion, and rarely *NRAS* Q61R) followed by a secondary alteration activating Wnt/β-catenin signaling, most commonly activating *CTNNB1* mutation or, less often, inactivation of *APC* (Figure 1A) [14,169].

Compared with DPN, DPNLMs are enriched with *TERT*p mutations, show pathogenic alterations in *CDKN2A* and SWI/SNF complex genes such as *ARID1A*, *ARID1B*, *ARID2*, *PBRM1*, and *SMARCA4*, have an overall higher tumor mutational burden, and are somewhat more likely to harbor *NRAS* and/or *APC* mutations (Figure 1) [169,170,171,172,173,174,175]. Interestingly, a recent study identified an *EGFR* driver mutation, an upstream activator of the MAPK pathway, in one case of DPNLM [172]. Alternate mutations were seen in Wnt/beta-catenin wild-type cases, with mutations in *EZH2*, *FAT1*, and *SETD2* that were predicted to indirectly modulate Wnt/β-catenin signaling [172].

A diagnostic role for chromosomal copy number aberrations in deep penetrating melanocytic neoplasms remains unclear. Comprehensive analyses have demonstrated that DPNLMs can harbor multiple CNAs [169,175]; however, these are not reliably detected in DPNLM by FISH or focused NGS panels [171,172]. Although one SNP-array-based study reported higher CNAs in DNPLMs as compared to DPNs, this observation could at least in part reflect incorporation bias, as CNAs were a component of their classification approach [175]. Thus, comprehensive approaches such as SNP array can detect CNAs in DPNLM, but additional rigorous objective evidence is necessary to establish clear criteria for CNA-based diagnostic classification of deep penetrating melanocytic neoplasms as benign, intermediate, or malignant. Furthermore, focused assays such as FISH may have limited sensitivity in this context [171].

#### 5.8.3. Clinical Features

WNT-activated DPMNs are often symmetric, darkly pigmented papules measuring less than 1 cm in the greatest dimension, most often presenting on the head and neck [9].

#### 5.8.4. Microscopic Features

**Figure 8:**
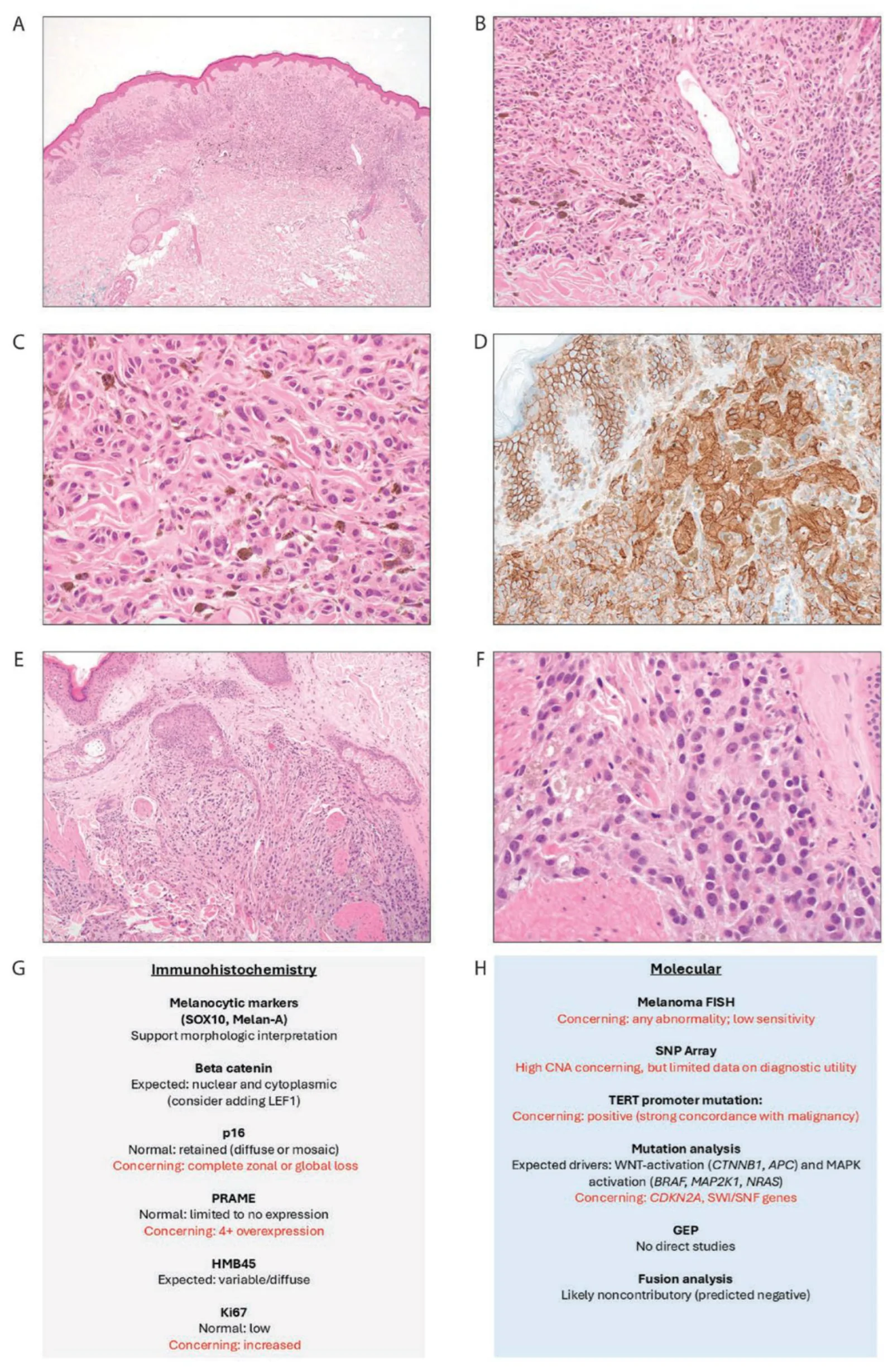
Deep penetrating nevus (Wnt-activated deep penetrating/plexiform melanocytoma) (**A**–**D**) and deep penetrating nevus-like melanoma (**E**,**F**). (**A**) Scanning magnification revealing an asymmetric predominantly intradermal melanocytic neoplasm. (**B**) Intermediate magnification reveals two populations of cells: epithelioid forms with abundant cytoplasm not showing maturation with dermal descent, and an associated cytologically banal component consistent with ordinary nevus. (**C**) High magnification reveals that the epithelioid forms contain abundant cytoplasm with pleomorphic nuclei and variably prominent nucleoli but without conspicuous mitotic activity. Numerous melanophages are intermixed. (**D**) Immunohistochemistry for β-catenin reveals nuclear and cytoplasmic expression in the epithelioid population with only membranous expression observed in the ordinary nevus component and in the overlying epidermis. (**E**) Recurrent deep penetrating nevus-like melanoma showing expansile growth. (**F**) High magnification reveals rhabdoid cytomorphology as well as numerous mitotic figures. Stains: (**A**–**C**,**E**,**F**) H&E, (**D**) DAB chromogen with hematoxylin counterstain. Magnification: (**A**) × 40 (**B**) ×200 (**C**,**D**) ×400 (**E**) ×100 (**F**) ×400. (**G**) Potential immunohistochemical (IHC) panel. (**H**) Molecular diagnostic tests that may be informative. As detailed in the text, results of ancillary IHC and molecular testing should be considered in the full context of findings for a given case.

Microscopically, WNT-activated DPMNs are characterized by a wedge-shaped predominantly dermal melanocytic lesion comprised of enlarged melanocytes (approximately twice the size of basal keratinocytes) with fine granular cytoplasmic pigment and scattered melanophages. Mitotic rate is typically less than 2 mitoses/mm^2^. A conventional/ordinary nevus is often identified [9] (Figure 8).

#### 5.8.5. Immunohistochemistry

A proposed immunohistochemistry panel is shown in Figure 8G. The presence of both common drivers may be detected using immunohistochemistry, with a *BRAF* V600E-specific antibody and one for β-catenin. Nuclear and cytoplasmic (active) patterning of β-catenin will be observed in the deep penetrating melanocytes as opposed to the membranous (inactive) pattern seen in ordinary nevomelanocytes [169]. One caveat is that nuclear and cytoplasmic β-catenin expression is typically seen in superficial dermal pigment-producing type A nevomelanocytes; however, membranous expression is observed upon maturation with descent into the dermis in ordinary nevi [169]. Thus, β-catenin immunohistochemistry should be interpreted with caution in superficial melanocytes.

#### 5.8.6. Differential Diagnosis

Deep penetrating nevus-like melanomas (DPNLMs) are often challenging to separate from DPN histopathologically but tend to be larger tumors with more nuclear atypia, increased mitotic activity, sheet-like growth, and more common involvement of subcutaneous tissue [170,171]. Additional molecular studies such as next-generation sequencing may be helpful (Figure 8H). Mutation testing can support the diagnosis of WNT-activated DPN/DPNM by confirming the presence of *CTNNB1* or *APC* mutations and further assess for additional malignancy-associated aberrations such as mutations in *TERT*p, *CDKN2A*, or SWI/SNF genes [169,170,171,172,173,174,175].

### 5.9. BAP-1-Inactivated Melanocytic Tumors/Melanocytomas

#### 5.9.1. Definition

BAP1-inactivated melanocytic tumors (BIMTs) are also classified as melanocytomas in the current *WHO Classification of Tumours* and are often biphenotypic with an associated precursor ordinary nevus [14].

#### 5.9.2. Molecular Pathogenesis

*BRAF* is the most common initiating driver mutation with superimposed inactivation of *BAP1* [176,177,178]. Less frequent initiating drivers include *NRAS* mutation and *RAF1* fusion [176,179,180,181]. As *BAP1* is a tumor suppressor, inactivation of both alleles is required to establish the BIMT phenotype [177]. Heterozygous copy number loss involving a variable extent of the short arm of chromosome 3 encompassing *BAP1* is typically seen in combination with an inactivating point mutation, resulting in loss of both functional copies of the gene [176]. In contrast, homozygous 3p21 loss appears to be uncommon [176]. In a recent case series with SNP array testing, BIMTs demonstrated 0–6 CNAs (median = 2), with the majority (15/19) harboring ≥2 CNAs. This finding contrasts with the five BAP1-inactivated melanomas, which showed a substantially higher CNA burden, ranging from 6–10 CNAs (median = 7) [176]. Mechanisms resulting in copy-neutral loss of heterozygosity, including uniparental disomy, have also been observed [177].

Point mutations that conserve nuclear expression of BAP1 but that result in a catalytically inactive protein may also be encountered [182]. These tumors retain the characteristic morphologic features [182]. *BAP1* FISH demonstrating copy number loss can serve as a diagnostic adjunct for confirmatory purposes in many cases, although this does not demonstrate inactivating events that do not involve copy loss in a subset of BIMTs, such as uniparental disomy or a “second hit” mutation [179].

#### 5.9.3. Clinical Features

While most BIMTs occur sporadically, some may present in the context of *BAP1* germline mutations (BAP1 Tumor Predisposition Syndrome). In addition to developing multiple BIMTs, these patients have an increased risk of cutaneous and uveal melanoma, mesothelioma, basal cell carcinoma, and renal cell carcinoma [178]. Additionally, potential associations with meningioma, cholangiocarcinoma, and hepatocellular carcinoma have been proposed [183,184]. As BIMTs may be the presenting tumor for this syndrome, the diagnostic report for BIMT should include a comment alerting the clinician to the possibility of a tumor predisposition syndrome that may warrant genetic evaluation.

Although the vast majority of BIMTs appear to be indolent [178], melanomas may rarely develop from these neoplasms [176].

#### 5.9.4. Microscopic Features

**Figure 9:**
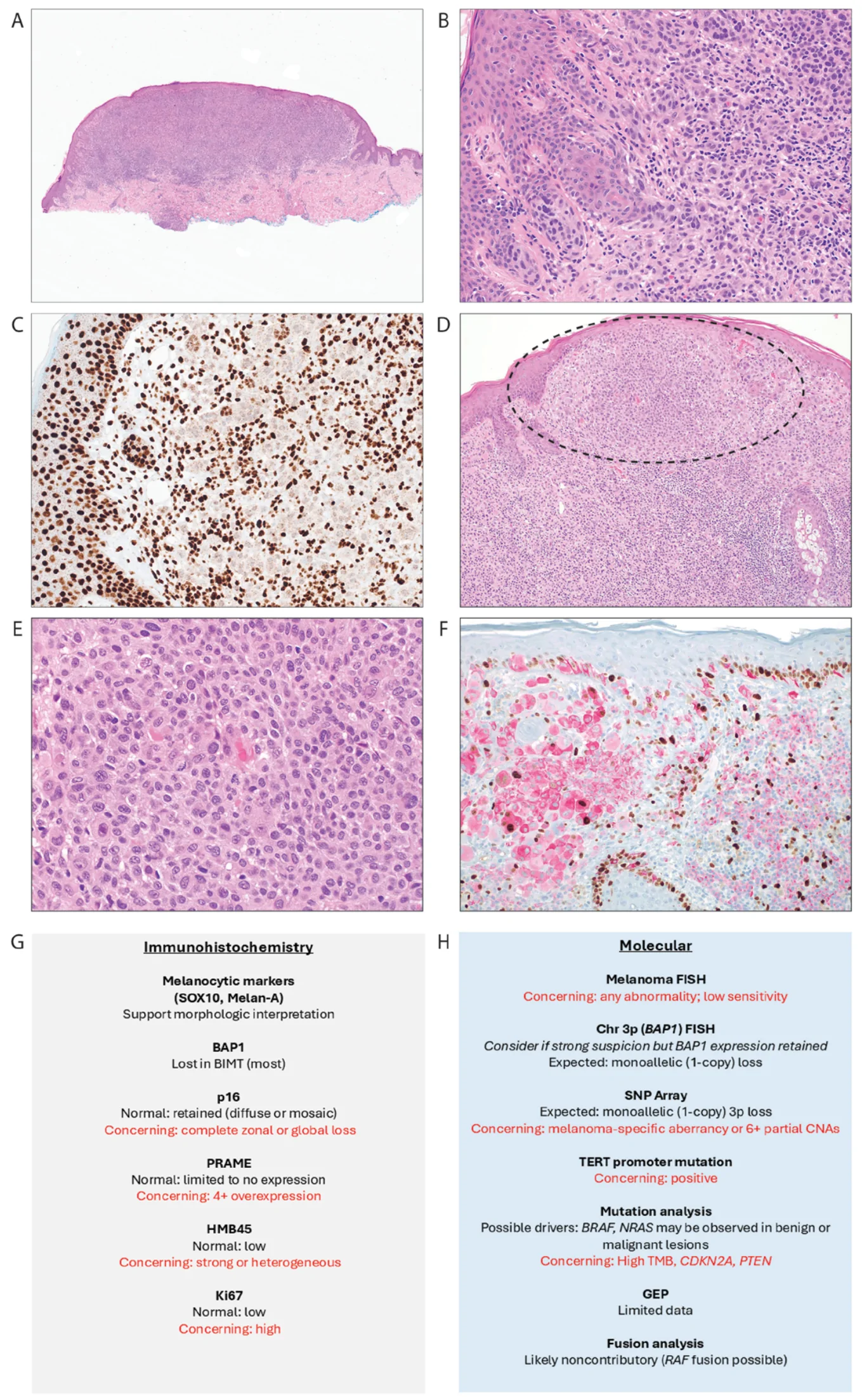
BAP1-inactivated nevus (melanocytoma) (**A**–**C**) and melanoma arising in a BAP1-inactivated nevus (**D**–**F**). (**A**) Scanning magnification reveals an exophytic lesion that is symmetric and densely cellular. (**B**) Intermediate magnification shows a biphenotypic compound melanocytic neoplasm containing an epithelioid component (upper right) as well as a cytologically banal component (lower left comprising junctional nests) consistent with ordinary nevus. There is an associated brisk lymphocytic infiltrate. (**C**) BAP1 immunohistochemistry reveals loss of expression in the epithelioid population (expression is retained in background inflammatory cells and ordinary nevomelanocytes). (**D**) BAP1-inactivated melanocytic tumor showing a superficial expansile nodule within a background of typical BAP1-inactivated nevus. (**E**) High magnification of the circled area reveals a monomorphic population of cells with a high mitotic rate. (**F**) A Ki67/MART-1 duplex immunostain showing a high proliferation index in melanoma cells and a low proliferation index in background BAP1-inactivated nevus. Stains: (**A**,**B**,**D**,**E**) H&E, (**C**) DAB chromogen with hematoxylin counterstain, (**F**) Ki67-DAB, MART1-Red, hematoxylin counterstain. Magnification: (**A**) ×40 (**B**,**C**) ×400 (**D**) ×100 (**E**) ×400 (**F**) ×200. (**G**) Potential immunohistochemical (IHC) panel. (**H**) Molecular diagnostic tests that may be informative. As detailed in the text, results of ancillary IHC and molecular testing should be considered in the full context of findings for a given case.

Histologic features such as epithelioid cytomorphology, well-defined cytoplasmic borders, and an associated brisk lymphocytic infiltrate correlate with BAP1 inactivation (Figure 9A–F). A precursor nevus can often be identified.

Rare progression to melanoma is usually characterized by outgrowth of a morphologically identifiable third component, often with higher-grade atypia and an increased mitotic rate [176].

#### 5.9.5. Immunohistochemistry

The most helpful immunohistochemical finding is the loss of nuclear BAP1 expression, although BIMTs with catalytically inactive BAP1 may show preserved protein expression. An immunohistochemical panel is summarized in Figure 9G.

#### 5.9.6. Differential Diagnosis

Morphologically, BAP1-inactivated melanocytomas may resemble Spitz nevi. The identification of *BRAF* or *NRAS* mutations helps to further exclude a lesion of Spitz lineage. There is also risk of misclassification as melanoma; confirmation loss of BAP1 by immunohistochemistry or molecular methods in the absence of overt malignant features helps to exclude melanoma arising in association with a nevus [9]. In cases with BIMT morphology but retained BAP1 protein expression, molecular modalities such as SNP array or BAP1 FISH can evaluate for the expected chromosome 3p (*BAP1*) monoallelic copy loss. As described above, SNP array can also demonstrate genomic instability in malignant lesions, although there may be a higher baseline rate of chromosomal copy number changes in benign/indolent BIMTs. Mutation testing may also reveal concerning features such as mutations in *TERT*p, *PTEN*, or *CDKN2A*. A proposed molecular testing strategy is summarized in Figure 9H.

### 5.10. Ocular Melanoma

#### 5.10.1. Definition

Ocular melanoma is characterized as melanoma arising from the conjunctiva or uveal tract [9].

#### 5.10.2. Molecular Pathogenesis

Ocular melanocytic neoplasms can be divided into two broad categories: conjunctival and uveal.

Conjunctival melanoma, unlike melanoma of the uveal tract (discussed below), shows a high degree of genetic overlap with conventional cutaneous melanoma. *BRAF* and *NF1* are the most frequently mutated genes, with the majority of *BRAF* mutations being the V600E variant, followed by *NRAS* (most commonly Q61R) [185,186]. *BRAF*, *NRAS*, and *NF1* mutations appear to be mostly mutually exclusive [187]. In some series, *NF1* mutations predominate [188]. Like cutaneous melanoma, a subset of cases is triple negative, without driver mutations in *BRAF*, *NRAS*, or *NF1* [188,189]. The driver mutations in conjunctival nevi mirror those seen in cutaneous nevi, with *BRAF* being most frequently mutated, followed by *NRAS* [190,191,192]. Although data are limited to two cases, conjunctival blue nevi likewise appear to harbor activating mutations in *GNAQ* [192]. Conjunctival melanomas usually display a UV signature with an intermediate to high tumor mutational burden [185,186,189]. *TERT*p mutations are seen in approximately 50% of cases [185,186]. Notably, *GNAQ*/*GNA11* mutations are absent in conventional conjunctival melanoma and are instead characteristic of uveal melanoma, discussed below. Conjunctival melanoma shows a diverse array of copy number variations that mirror those seen in cutaneous melanoma [186,187].

Most uveal melanomas are genetically distinct from conjunctival melanomas. Approximately 50% of patients eventually develop metastatic disease, preferentially to the liver [193]. However, anterior (iris) uveal melanomas, which are far less common than posterior (choroid and ciliary body) melanomas, have a significantly better prognosis [194]. While anterior uveal tumors have a higher tumor mutation burden (TMB) and a UV signature similar to conjunctival melanoma, posterior tumors lack a UV signature and have a relatively low tumor mutational burden [193]. Overall, approximately 80–90% of uveal melanomas harbor mutually exclusive mutations in either *GNAQ* or *GNA11* regardless of location [80,102]. *CYSLTR2* L129Q and *PLCB4* D630Y, which are mutually exclusive with *GNAQ* and *GNA11* mutations, account for most remaining cases [85,193,195]. Secondary mutations include (nearly) mutually exclusive mutations in *EIF1AX, SF3B1*, and *BAP1;* these inform risk, with *BAP1* mutations strongly associated with metastasis and melanoma-specific mortality. *SF3B1* mutations are associated with intermediate risk with delayed metastasis, and *EIF1AX* associated with a low risk of progression [196,197]. Driver and canonical secondary mutations are thought to occur early in tumorigenesis (rather than secondary mutations representing a later progression event), which may explain the lack of improvement in overall survival outcomes despite evolution in primary treatment [198]. Several recurrent copy number variations are found in uveal melanoma, including gain of 8q, gain of 6p, monosomy 3, and others, and also inform risk of progression [199].

Uveal melanomas may be separated into two genetic classes (class 1 and class 2, low- and high-risk, respectively) using a 15-gene expression panel (GEP) [200,201]. Genes upregulated in class 2 include *CDH1, ECM1, HTR2B*, and *RAB31*; downregulated genes include *EIF1B, FXR1, ID2, LMCD1, LTA4H, MTUS1, ROBO1*, and *SATB1*. Three control genes are also included. Class 2 GEP is associated with copy number variations including monosomy 3 and 8q gain, as well as BAP1 mutation. Gene expression analysis has emerged as a well-validated major prognostic marker in uveal melanoma [200,202,203,204]. One recent study, however, instead found tumor diameter to be the most important predictor of metastasis [205]. When combined with *PRAME* RNA expression, the 15-GEP is enhanced, with class 2/*PRAME*-positive patients having a 5-year metastasis-free survival of 44.8% [206,207]. Addition of tumor diameter further improves prognostic value, and the combination of GEP class, *PRAME* RNA expression, and tumor diameter currently provides the best prognostic accuracy [182].

Performance of the GEP classifier assay may vary significantly between historical cohorts and contemporary treatment populations. For example, recent data show that GEP-predicted metastasis-free survival may be worse than observed in some contemporary cohorts, with larger class 2 tumors having a greater metastatic risk [208]. Additionally, although class 1 may be further separated into class 1A and 1B, this distinction may not be relevant, contrary to prior reports [205].

While treatment of uveal melanoma is generally based on tumor size, further risk stratification incorporating GEP results, *PRAME* RNA expression status, and selected CNAs can be used to tailor ongoing clinical surveillance [209]. Risk category is assigned according to the highest risk factor identified. All patients are recommended to undergo imaging for signs or symptoms concerning metastatic disease. Patients with low-risk tumors (class 1A or class 1/*PRAME*-negative; tumors containing disomy 3, 6p gain, and/or *EIF1AX* mutation; AJCC T stage T1) are recommended to undergo surveillance imaging every 12 months for 5 years. For patients with intermediate-risk tumors (class 1B or class 1/*PRAME*-positive; tumors containing an *SF3B1* mutation; T2-T3), surveillance imaging is recommended every 6–12 months for 10 years. Patients with high-risk tumors (class 2; tumors containing monosomy 3, 8q gain, and/or somatic *BAP1* mutation; T4) are recommended to undergo imaging every 3–6 months for 5 years, followed by imaging every 6–12 months for an additional 5 years. Because the liver is the most common site of metastasis, surveillance should include liver imaging, preferably with MRI. The affected eye should also be followed at regular intervals after definitive treatment [209].

#### 5.10.3. Clinical Features

Ocular melanomas have a heterogeneous clinical appearance ranging from macular to nodular, amelanotic to darkly pigmented, and circumscribed to irregular. Ancillary imaging studies may be helpful when there is a high index of clinical suspicion [210].

#### 5.10.4. Microscopic Features

**Figure 10:**
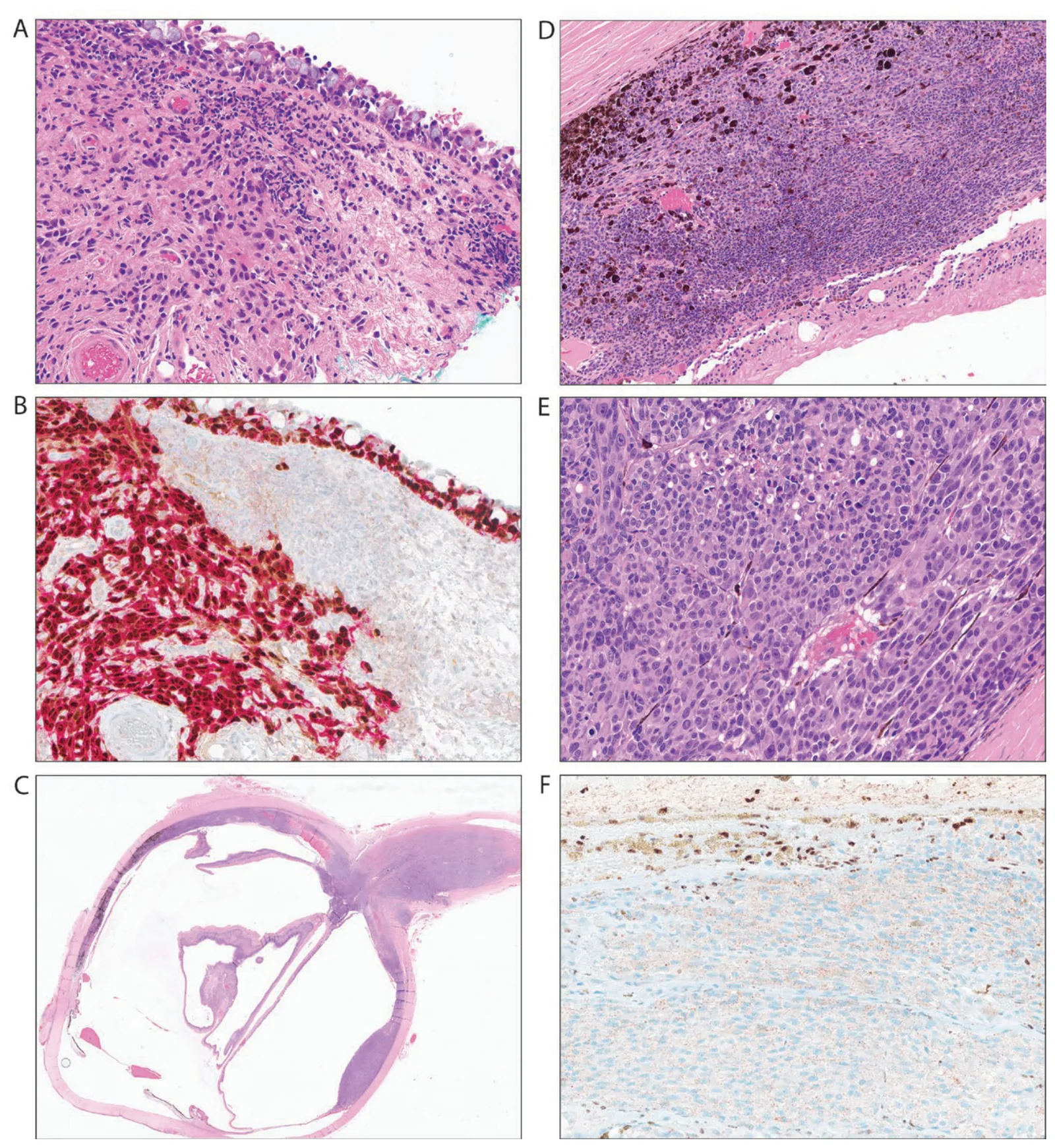
Ocular melanoma. (**A**) Conjunctival melanoma showing an atypical junctional component consistent with melanoma in situ (“primary acquired melanosis” with severe atypia) as well as a cytologically atypical population of melanocytes in the substantia propria. (**B**) A PRAME/MART-1 duplex stain reveals diffuse nuclear PRAME labeling (4+). (**C**) Scanning magnification image of a globe enucleation specimen showing circumferential involvement by choroidal/ciliary body melanoma with invasion into the optic nerve. (**D**) Intermediate magnification image showing a cellular tumor within the choroid with numerous associated melanophages. (**E**) High magnification reveals predominantly epithelioid melanocytes with enlarged nuclei and prominent nucleoli. Occasional spindle cell forms with a blander appearance can be seen at the periphery of the tumor (lower right). (**F**) BAP1 immunohistochemistry reveals diffuse loss of expression in tumor cells, while intact staining is seen in infiltrating immune cells. Stains: (**A**,**C**–**E**) H&E, (**B**) PRAME-DAB, MART1-Red, hematoxylin counterstain, (**F**) DAB chromogen with hematoxylin counterstain. Magnification: (**A**,**B**) ×400 (**C**) ×8 (**D**) ×200 (**E**,**F**) ×400.

Histopathologic features are similar to those of melanoma in the skin or mucosal sites. There is often an in situ component with nested, lentiginous, and/or pagetoid growth. The subepithelial component demonstrates features typical of malignancy such as pleomorphism, mitotic activity, and absence of maturation [9] (Figure 10).

#### 5.10.5. Immunohistochemistry

Ocular melanoma demonstrates similar immunohistochemical findings to melanoma of cutaneous sites. Melanocytes are positive for markers of melanocytic lineage. In conjunctival melanomas, PRAME positivity and loss of p16 favor malignancy in the appropriate clinical and histopathologic contexts [9]. PRAME protein expression by immunohistochemistry in uveal melanoma appears to correlate with *PRAME* RNA expression status [211]. Additionally, there is evidence that BAP1 expression by immunohistochemistry may be a reliable surrogate for *BAP1* genetic testing [212].

#### 5.10.6. Differential Diagnosis

Similar to cutaneous melanoma, the differential diagnosis includes nevi and melanocytomas. Metastatic melanoma must also be excluded. Of note, most subtypes of nevi and melanocytomas mentioned in the above sections have been described within the ocular structures [9].

## 6. Diagnostic Molecular Testing of Melanocytic Lesions

### 6.1. Overview

**Figure 11:**
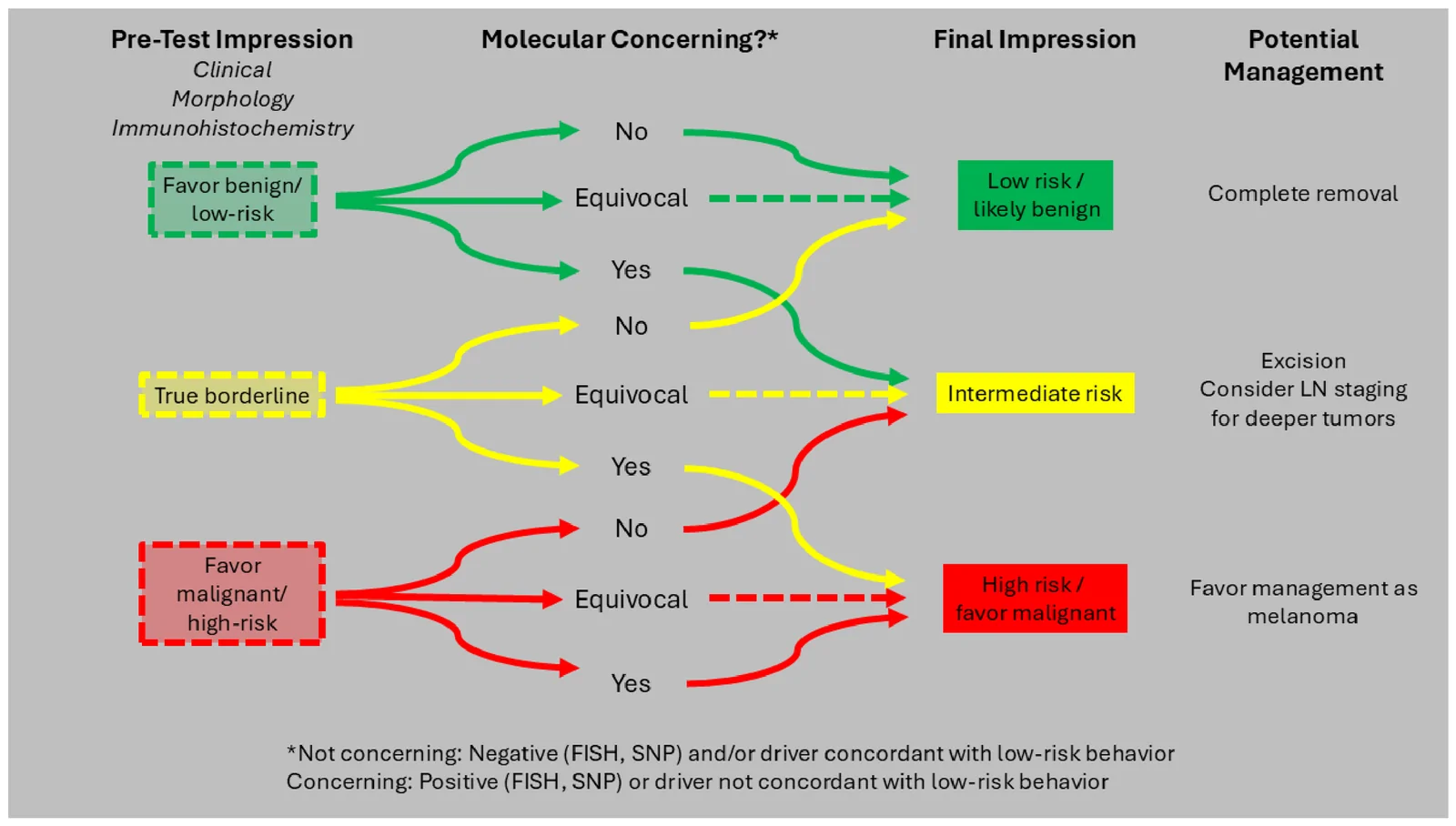
Schematic for incorporation of molecular testing results into melanocytic diagnosis.

The vast majority of melanocytic neoplasms can be accurately diagnosed by histomorphology and immunohistochemistry alone. Molecular tests are reserved for a small fraction of lesions, particularly ambiguous lesions in which both benign and malignant entities are in the diagnostic consideration [11,12,13]. In such cases, additional data on molecular features of melanocytic neoplasms can provide an essential component of comprehensive expert analysis alongside morphology, immunophenotyping, and clinical details. An algorithmic approach for incorporating molecular test results into the final diagnosis has been described [11], with a simplified/summary version shown in Figure 11. Importantly, molecular diagnostic test results should not generally be considered sufficient for a benign or malignant diagnosis without considering the full context of the case. Molecular testing modalities include FISH, comparative genomic hybridization (CGH)/SNP array, next-generation sequencing (NGS), and gene expression profiling (GEP). Each modality has distinct strengths, limitations, and clinical utility (Table 1) [11,12,13].

### 6.2. Fluorescence in Situ Hybridization (FISH)

FISH utilizes fluorescent probes to detect chromosomal copy number aberrations in select hotspots frequently affected in melanoma [11,12,13]. The original 4-probe panel targeting *RREB1* (6p25), *MYB* (6q23), centromere 6 (cep 6), and *CCND1* (11q13) was found to have a sensitivity of 86.7% and specificity of 95.4% for identifying melanoma [213]. The Gerami criteria, most widely used in the literature, require at least 30 lesional nuclei for testing. Results are positive when the percentages of lesional nuclei containing >2 copies of *RREB1*, >2 copies of *CCND1*, copies of *RREB1* exceeding Cep6, or copies of *MYB* falling short of Cep6 reach certain thresholds [11,12].

A more commonly used second-generation panel includes the additions of *CDKN2A* (9p21) and *MYC* (8q24), in which homozygous deletion of *CDKN2A* or >2 copies of *MYC* in certain percentages of lesional cells are considered positive results. The additions demonstrated improved sensitivity and specificity at 94% and 98% compared with the original 75% and 96%, respectively, in the same validation cohort by Gerami et al. [120].

It is worth noting that validation cohorts in most studies, including the seminal studies by Gerami et al., consisted mostly of unequivocal “gold standard” melanomas and benign nevi. A few studies have examined the utility of FISH in diagnosing ambiguous melanocytic neoplasms and demonstrated potential for much lower sensitivity and specificity, ranging from 43–100% and 33–100% [213,214,215]. In a series of 34 ambiguous melanocytic tumors, Tetzlaff et al. reported a sensitivity of 50% and specificity of 87.5% compared with histopathologic diagnosis [216]. Similarly, in a series of 22 melanocytic tumors, including 12 ambiguous ones, Gaiser et al. reported a sensitivity of 60% and specificity of 50% [214]. Most of the ambiguous lesions tested in these studies were atypical Spitz tumors (Spitz melanocytomas), and the studies in general showed that FISH in combination with expert histopathologic review improves diagnostic accuracy compared to either modality alone.

FISH has important limitations. Notably, loss of *MYB* may not be predictive of aggressive disease in the specific context of atypical Spitz tumors [217], in contrast to conventional melanoma, for which chromosome 6q loss has been correlated with worse outcomes [218]. Thus, for Spitz neoplasms, only the second-generation panel should be used and should always be interpreted in conjunction with histopathologic correlation and possibly other molecular results [122,219]. In some melanomas, a substantial number of CNAs may involve genomic loci not probed by FISH [220]. An additional source of false-negative results is the potential for large FISH probes to miss small homozygous deletions of *CDKN2A* [220], as discussed further below.

Although FISH has overall robust specificity, false positive results are possible. Tetraploidy can create the impression of chromosomal gains if not carefully accounted for. Unlike SNP array, FISH does not distinguish whole chromosome gains from focal oncogene gain for loci not accompanied by a centromere probe (e.g., *CCND1, MYC)* [220]. Scoring FISH for melanocytic lesions also requires a degree of expertise [11].

Although FISH has limitations, it remains a valuable option in particular contexts. In particular, the sample size requirement is very low (for example, scoring of 30 tumor cells) and lesional cells can be selected for scoring among other cell types. Thus, FISH may remain a viable testing option for samples that do not qualify for other molecular testing due to smaller size, limited tumor purity, or nucleic acid quality [11,12].

Considered together, these findings indicate that FISH has diagnostic utility in specific contexts. While a positive result strongly supports melanoma, a negative result does not exclude malignancy [214,216,219,221,222], and limitations of this test, including potential false positives and negatives, must be kept in mind. In addition, as with all ancillary tests, FISH should not be used as a standalone or decisive test for borderline lesions, particularly atypical Spitz neoplasms.

### 6.3. Comparative Genomic Hybridization (CGH)/Single Nucleotide Polymorphism (SNP) Array

CGH)/SNP array, similar to FISH, detects CNAs. Unlike FISH, it hybridizes labeled tumor DNAs to microarrays containing probes distributed across the entire genome [11,13].

Traditional array-based CGH calculates the ratio of signal intensities between tumor and reference DNA from normal human tissue to reveal chromosomal gains and losses. However, this traditional approach did not detect copy-neutral loss of heterozygosity (LOH), a frequent occurrence in melanoma, because no net change in copy number occurs [11].

**Figure 12:**
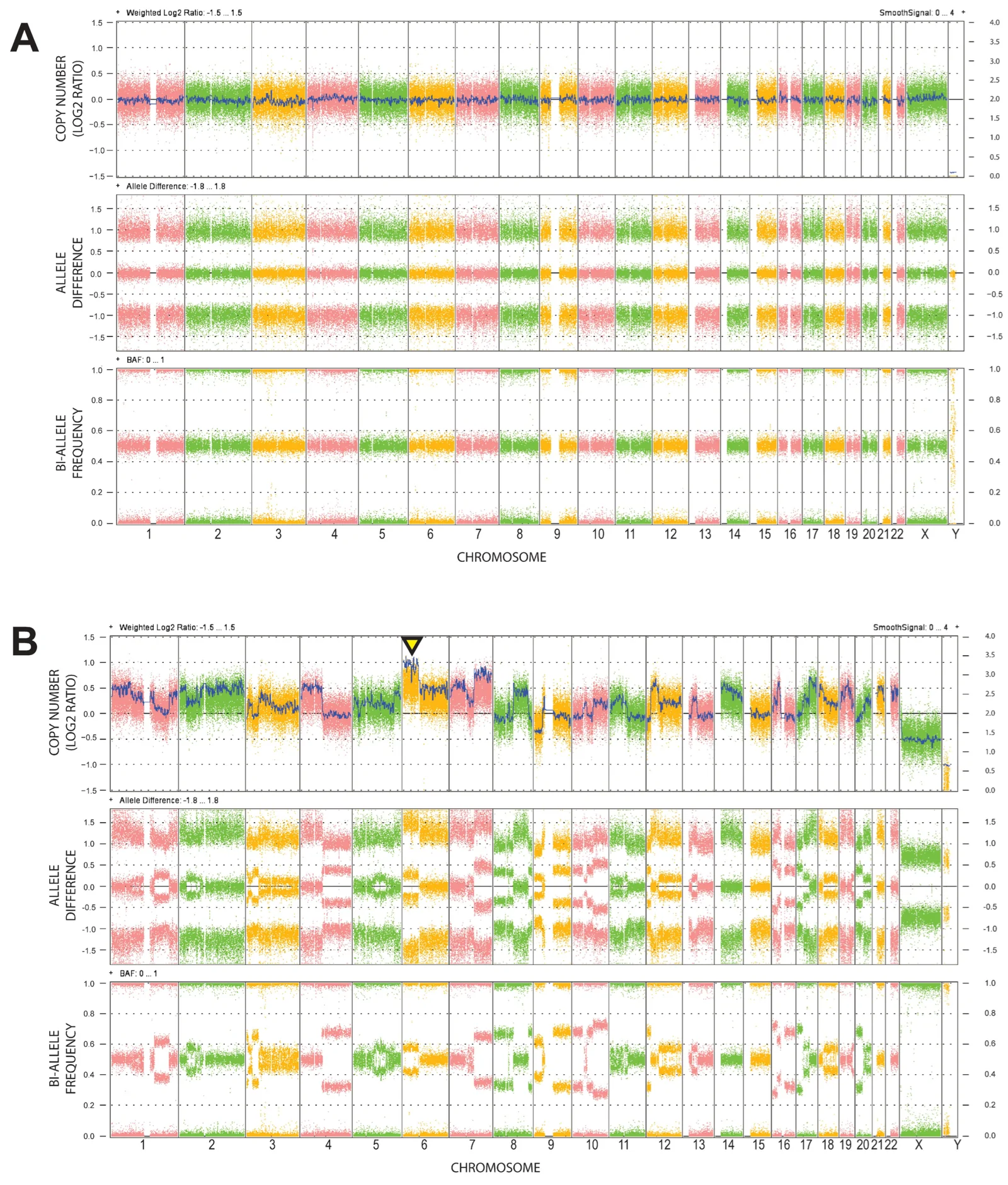
Example SNP array results. (**A**) Normal female genome with 2 copies of all chromosomes except X (upper row) and the expected distribution of allele frequencies (lower 2 rows). (**B**) Example results from melanoma with marked genomic instability resulting in numerous copy number alterations, including gain of the oncogene RREB1 at 6p (yellow arrowhead). Chromosomes are differentially colored for visual clarity.

More recently developed SNP arrays resolved this issue by utilizing SNP-specific probes to define allele frequency and copy number quantification relative to a reference dataset (Figure 12). To perform this analysis on the highly fragmented DNA that results from formalin cross linking in clinical samples, molecular inversion probes, which require only very short intact DNA sequences (~40 bp), may be used for hybridization, eliminating the need for enzymatic restriction and PCR amplification of genomic DNA required by other platforms. To further reduce noise, an engineered surrogate sequence tag, rather than tumor DNA itself, hybridizes with the microarray, and an enzymatic digestion step removes unused probes and template DNA [11,223,224].

Early validation studies focused on the test’s ability to distinguish unequivocal melanomas from benign nevi. In the seminal study by Bastian et al., 96.2% of melanomas demonstrated chromosomal aberration, with a mean of 7.5 aberrations, compared to 13% of benign nevi, all of which were Spitz nevi harboring an isolated 11p gain [225]. Subsequent studies reported 83–95% of melanomas containing multiple CNAs, whereas 95–100% of benign nevi contained no CNAs (Figure 12B,C) [223,226,227].

The interpretation of these assays for diagnostically ambiguous or borderline melanocytic neoplasms is more nuanced because such cases can lack the clear dichotomy in CNA burden observed between benign nevi and melanomas. A study by Alomari et al. demonstrated a stepwise increase in average CNA burden, progressing from 0 in nevi, 0.6 in atypical nevi, 2.8 in ambiguous tumors, to 18.1 in melanomas [32]. By comparing CNA burden in tumors with and without adverse events, the authors proposed a threshold of greater than three CNAs as a positive result, as no neoplasm associated with adverse events showed less than or equal to 3 CNAs [32].

A meta-analysis by Ebbelaar et al. evaluated CNA thresholds to distinguish benign/intermediate from malignant melanocytic neoplasm and demonstrated an 85% sensitivity and 84% specificity using a cutoff equal to or greater than three CNAs, and an 81% sensitivity and 91% specificity at a cutoff equal to or greater than four CNAs [33]. A 2026 evidence-based review by the Cancer Genomics Consortium (CGC) Working Group systemically reviewed published studies from 1998–2022 and proposed an algorithm for melanocytic neoplasms indefinite for malignancy using greater than three CNAs as the cutoff value as support for malignancy [228]. However, awareness of potential context-dependent limitations of this assay is also essential, including the possibility that a higher threshold may be appropriate for BIMTs and potentially reduced sensitivity for malignancy in certain contexts such BNLM or DPNLM.

The pattern of CNAs also carries diagnostic significance. For example, the presence of homozygous *CDKN2A* deletion is suspicious for melanoma even when present as a single CNA event. There is similar significance for partial chromosomal gains involving *CCND1*, *RREB1*, and *MYC*. In contrast, gains and losses of whole chromosomes, such as seen in proliferative nodules within congenital nevi, are not counted as high-risk features [11].

Chromothripsis is the phenomenon of an area of chromatin (involving one or several chromosomes) displaying numerous amplifications and/or deletions as a result of a localized catastrophic DNA damage event. Although chromothripsis has been associated with worse prognosis in definitive melanoma, ref. [229] in the context of diagnostically ambiguous lesions, this finding is not correlated with aggressive disease [32,33]. For purposes of CNA quantitation, chromothripsis is considered a single event rather than enumerating every focal gain or loss [11,32,33].

A limitation of SNP arrays is the inability to detect mutations or gene rearrangements. CNAs can be suggestive of oncogene mutations, especially chromosome 11p gain correlating with activating *HRAS* mutation [105,230]; however, direct mutation testing is generally preferable. Similarly, although a truly balanced chromosomal rearrangement would be copy-neutral, gene fusion events are often associated with CNAs transecting the gene body of one or both fusion partners [231,232]. Although this finding is strongly suggestive of an oncogenic rearrangement, RNA-based approaches are optimal to confirm the presence of a functional expressed fusion transcript [11,12,13].

Technical limitations include the potential for false negatives related to tumor purity, including potential sampling of background precursor nevus. In addition, SNP arrays require substantial cost, time, and input of tumor material [11,12,13].

In summary, SNP arrays provide genome-wide characterization of CNAs to allow for identification of oncogene amplification, tumor suppressor copy loss, and quantitation of chromosomal instability. Robust evidence supports a threshold of 3–4 partial chromosomal gains or losses as evidence for malignancy in many scenarios, although this may be less reliable in some contexts.

### 6.4. TERT Promoter (TERTp) Mutations

The telomerase reverse transcriptase (*TERT*) gene, located on chromosome 5p15.33, encodes the catalytic subunit of telomerase responsible for maintaining telomere length during cell division [233,234]. Although *TERT* is silenced in most somatic cells, it is reactivated in up to 90% of human cancers [235,236]. One of the mechanisms is through mutations in the *TERT* promoter (*TERT*p), leading to increased *TERT* expression, cellular immortality, and disease progression across a wide range of cancers, including melanoma [233,234]. Hotspot *TERT*p mutations occur at positions c.−124C>T, c.−124_−125CC>TT, c.−138_-139CC>TT, and c.−146C>T [237].

A study by Shain et al. identified *TERT*-p mutations in 77% of intermediate melanocytic neoplasms and melanoma in situ and none in wholly benign neoplasm [29]. Subsequent studies have found 30–80% of non-acral cutaneous melanomas harbor *TERT*-p mutations depending on the subtype [174,237,238,239,240]. This contrasts with 0–6% in benign and dysplastic nevi [174,237,239].

The role of *TERT*-p mutations in malignant progression is complex. *TERT*-p mutations can be detected alongside *NRAS* mutations in intermediate lesions (lacking overt malignant progression). However, the co-occurrence of *TERT*-p mutation and *BRAF* V600E mutation is relatively restricted to melanoma, with *TERT*-p mutation at low levels or absent in *BRAF*-mutant nevi [23,29]. These mutations may thus contribute to the progression of benign nevi to melanoma in situ or intermediate melanocytic lesions or may give rise to intermediate lesions that completely bypass the benign phase [29].

*TERT*-p mutations are particularly useful in differentiating between atypical Spitz tumor and spitzoid melanoma, atypical deep penetrating nevus (DPN) and melanoma, and dysplastic nevus and melanoma, especially those arising in sun-exposed skin [121,174,175,241]. They are less informative for the blue melanocytic lesion group [92,174].

The ASDP Appropriate Use Criteria committee has classified the diagnostic utility of *TERT*-p mutations as “uncertain appropriateness.” This limitation may reflect the earlier emergence of *TERT*-p mutations in melanomagenesis, leading to overlap with melanoma in situ and dysplastic nevi [12,29]. *TERT*-p mutation testing may also have relatively lower negative predictive value, as a substantial proportion of melanomas, particularly those arising in acral or mucosal sites, do not harbor *TERT*-p mutations [174,242]. In summary, detection of *TERT*-p mutation can raise concern for melanoma, but the specificity and sensitivity of this finding for malignancy is likely to be heavily context-dependent.

### 6.5. Next-Generation Sequencing (NGS)

NGS approaches can be divided into two broad assay types: DNA-based sequencing and RNA-based sequencing.

DNA-based NGS approaches can range from small panels of oncogenes to pan-cancer panels of over 1000 genes and even up to exome-wide or genome-wide approaches [243,244]. Depending on the approach, DNA-based NGS may detect driver mutations, passenger mutations, and TMB, as well as infer CNAs through read-depth analysis. Driver mutations are characterized by recurrent mutations at well-recognized hotspots, mutual exclusivity with other driver mutations, and conferring a selective growth advantage. Driver mutations in melanocytic neoplasms can be either oncogenic point mutations or insertions/deletions. Importantly, NGS can shed light on how far along a lesion has evolved in the tumor progression pathway. For example, in a lesion arising on the low-CSD pathway, NGS can simultaneously detect driver mutations in the MAPK pathway, early secondary events such as *TERT*-p mutations, progression events such as *CDKN2A* homozygous inactivation, and late-stage mutations such as *PTEN* loss or *TP53* mutations [12,29].

Genes commonly found in DNA-based NGS panels include initiating driver genes such as *BRAF, NRAS, NF1, KIT, HRAS, GNAQ, GNA11*, and *MAP2K1* (in-frame deletions). Of these, detection of *BRAF* mutations may also support management with BRAF/MEK inhibitors if indicated for melanoma. Panels may also include genes such as *CTNNB1* and *BAP1*, whose mutations may co-occur with the MAPK pathway driver mutations. Genes involved in tumor progression and invasion, including *TERT*p, *ARID1A, ARID1B, ARID2*, and *CDKN2A*, as well as genes associated with later progression events, such as *PTEN* and *TP53*, are also commonly included in comprehensive panels but may not be a component of small oncogene-focused panels [243,245,246,247,248,249,250,251]. Certain DNA-based panels are designed to detect gene rearrangements, for example, by targeting intronic regions typically involved in the rearrangements of interest [252]; however, such panels are uncommon, and RNA-based approaches are more frequently used for this purpose, as described below. Whole genome sequencing can directly demonstrate rearrangements, mutations, and CNAs across the genome; however, this approach is rarely used for clinical testing of melanocytic tumors due to cost and input requirements.

Melanomas tend to carry a higher TMB than benign and intermediate lesions. However, substantial overlap in TMB ranges between these categories results in limited diagnostic utility of this observation in practice. A recent study by Vergara et al. developed a bivariate genomic classifier that combines non-*BRAF* and non-*NRAS* coding mutational load and noncoding mutational load and achieved an AUC of 95–96% with >80% sensitivity and >95% specificity. The model demonstrated particular utility among 36 indeterminate tumors arising in the low-CSD pathway that lacked additional pathway-defining genomic alterations. Eight (22%) were identified as having high probability of melanoma. Seven of these eight had negative or non-aberrant results in other molecular assays (e.g., FISH, CGH), suggesting that mutational load analysis may identify melanomas that may be missed by chromosomal-based assays, as point mutations and UV-signature passenger mutations accumulate before alterations at the chromosome level [31]. However, the investigative arm of this study included only classic nevi and melanomas with specific exclusion of subtypes, including Spitz, BAP1-inactivated tumors, and PEMs. Moreover, malignant counterparts of nonconventional nevi (Spitz melanoma, melanoma ex blue nevus, etc.) were not included; thus, performance for UV-independent melanoma subtypes remains unclear.

RNA-based NGS panels primarily identify kinase gene fusions, which are particularly relevant for Spitz neoplasms and triple wild-type melanomas. The targeted genes fall into the broad categories of receptor tyrosine kinase fusions (e.g., *ALK*, *ROS1*, *NTRK1*/*3*, *MET*, *RET*) and serine/threonine kinase fusions (e.g., *BRAF*, *RAF1*, *MAP3K8*) [124,253]. Optimally, panels also detect other diagnostically relevant fusions, such as those associated with clear cell sarcoma (e.g., *EWSR1::ATF1)* or other tumors with melanocytic differentiation (e.g., *MITF::CREM, ACT::MITF, CRTC1::TRIM11, MED15::ATF1*) [9,12]. Anchored multiplex PCR, for example, the Archer FusionPlex platform, employs a single-gene primer to allow for detection of fusions with both known and novel 5′ partners. Hybrid capture NGS approaches can also detect fusions based upon targeting of only a single partner. Such approaches are particularly useful for Spitz neoplasms which are characterized by a diversity of fusion partners. By contrast, multiplex PCR-based approaches require primers to target both 5′ and 3′ partners [12,252].

Limitations of NGS are similar to those of SNP array, including significant cost and tumor input requirements. CNA by NGS tends to be less robust than for SNP array, especially in the context of smaller panels.

In summary, NGS testing is highly useful for defining the class of a melanocytic lesion by identifying founding oncogenic drivers (Figure 1A). This can be particularly informative when the driver event explains a morphologic finding that would otherwise be concerning, or conversely when morphologic/molecular discordance raises suspicions. DNA-based NGS can distinguish melanomas from its borderline precursors by detecting secondary or progression events, although the relative incidence of some mutations in nevi vs. melanoma may be unclear, and algorithmic interpretation is less straightforward than for SNP arrays. TMB may also provide information, but the diagnostic utility of this metric is currently investigational and will likely be context-dependent. In contrast, RNA-based NGS is best suited for identifying the driver in fusion-associated Spitz neoplasms, PKC-fusion blue nevi, and some triple wild-type melanomas; beyond this, RNA-based fusion discovery NGS does not provide further insight regarding the benign or malignant nature of a melanocytic neoplasm. Thus, NGS approaches are often paired with an assay focused on risk stratification (such as SNP array) to provide more comprehensive molecular characterization.

### 6.6. Gene Expression Profiling

There are multiple commercially available gene expression profiling assays available for clinical use, each of which target varying numbers of genes and loci [254,255,256]. These assays can be divided into two broad categories: diagnostic (distinction of nevi from melanoma) and prognostic (e.g., estimating risk of sentinel lymph node involvement for melanoma) [12].

Diagnostic gene expression profiling assays draw upon large-scale expression studies comparing melanoma and nevi to assemble focused panels of genes predicted to be most informative in this distinction. One commercial diagnostic assay examines expression of 23 genes from tumorigenic, immune, multifunctional/miscellaneous, and housekeeping gene sets and weights the resulting expression results to generate a numerical score indicating malignant, borderline, or benign diagnosis [257]. Reported sensitivity and specificity have varied substantially across studies (62–93% and 81–96%, respectively [11,215,257,258,259,260,261]. In addition, there has been limited evaluation of challenging melanocytic lesions and malignant counterparts to unconventional nevi (e.g., Spitz melanoma) [11].

Multiple prognostic assays for cutaneous and uveal melanoma are based upon gene expression panels [11]. Gene expression panels for uveal melanoma (described in greater detail above) are in routine use. For cutaneous melanoma, while there are some promising preliminary data from studies assessing the utility of these assays, the general consensus among multiple working groups is that there is insufficient evidence to support their use in clinical practice with one notable exception [15,262]. The National Comprehensive Cancer Network consensus statement cites data from the MERLIN_001 trial to support gene expression profiling in conjunction with shared decision-making when considering sentinel lymph node biopsy in patients with pathologic stage pT1b or pT2a melanoma. This recommendation applies to patients with an overall risk of metastasis of <10% as predicted by the assay [15]. Limitations of the current landscape of gene expression profiling include: superior performance of established parameters such as Breslow depth, ulceration, and sentinel lymph node status; high cost to patients; and potential for delayed or inappropriate treatment if established NCCN guidelines are not followed. Thus, these assays are considered for cases where standard management recommendations are less definitive [262,263,264,265].

## 7. Direct Comparisons of Molecular Diagnostic Tests

Several large studies have systematically evaluated performance of clinically available molecular diagnostic tests by direct comparison against other tests or a gold standard:

Minca et al. compared a targeted FISH (panel 6p25 (*RREB1*), 11q13 (*CCND1*), 8q24 (*MYC*), and 9p21/*CEP9*) and GEP to both conventional and challenging lesions. For conventional lesions, FISH demonstrated 93% sensitivity and 100% specificity, whereas GEP had lower sensitivity (62%) but robust specificity (95%). For challenging lesions, FISH displayed 70% agreement with expert consensus diagnosis (56% sensitivity, 83% specificity), and GEP displayed 64% agreement (52% sensitivity, 80% specificity) [260].

Reimann et al. examined challenging cases relative to expert consensus diagnosis and observed limited sensitivity for FISH (61%) but with high specificity (100%), whereas GEP had lower sensitivity (50%) with reasonable specificity (93%). For selected cases, SNP array was performed and displayed better correlation with morphologic interpretation than FISH and GEP [215].

Carter et al. compared SNP arrays with “virtual FISH” (i.e., predicted FISH results based on SNP array findings) and observed for borderline tumors that FISH had only 61% sensitivity and 84% specificity, with SNP array considered as the gold standard. Discordances arose when (1) copy number alterations did not correspond to FISH probe loci and (2) when there were whole-copy number changes, such as for chromosome 11 (including *CCND1*), that would be considered positive by FISH but negative by SNP array [220]. Comparing with real (not virtual) FISH, they also observed small 2nd deletions of *CDKN2A*, resulting in homozygous losses that were detected by SNP array but not FISH [220], potentially related to failure of FISH probes to identify small homozygous *CDKN2A* deletions, as previously reported for Ewing Sarcoma and mesothelioma [266,267].

Castillo et al. studied intertest agreement for FISH, SNP, and GEP in challenging cases (without a defined gold standard). Excluding equivocal results, they observed 70% agreement between FISH and GEP and 78% agreement between GEP and SNP [268].

Regmi et al. compared GEP with FISH or SNP array in diagnostically ambiguous melanocytic lesions. A final diagnosis was established by expert consensus. Agreement with the final diagnosis was 77% for FISH, 82% for GEP, and 100% for SNP array [48].

There are important caveats for these comparisons. Expert consensus diagnosis has inherent limitations as a gold standard, and there can be additional confounding effects due to incorporation bias for those studies in which the diagnosis was made with knowledge of test results being evaluated. There are challenges with meaningful incorporation of intermediate/equivocal test results into analyses [269]. Methodological differences (e.g., variability in *MYC* and *MYB* inclusion among FISH studies, or definition of a positive result for SNP array) also limit comparisons across studies. With these limitations in mind, general conclusions across studies are that SNP array displays the strongest performance, with relatively less reliability for FISH and GEP tests.

## 8. Clonality Testing to Distinguish Metastases from New Primary

Molecular testing can have diagnostic utility in scenarios beyond a determination of nevus vs. melanoma. Patients previously diagnosed with melanoma can develop additional lesions, which may represent cutaneous metastasis or an additional primary melanoma [12]. The distinction between the two has significant implications on staging and treatment. While most cases are reliably distinguished by morphologic and clinical assessment [270], some remain ambiguous.

As described above, melanocytic neoplasia is initiated by a driver mutation, most commonly *BRAF* V600E or *NRAS* Q61R/L [23,55]. Additional mutations such as copy number loss of *CDKN2A* (9p21) are associated with progression to melanoma [271]. The initiating mutation, as well as most mutations acquired throughout melanoma progression, are retained in metastatic lesions [272,273,274]. Occasionally, metastatic melanomas display additional mutations distinct from the primary tumor [273,275,276]. By contrast, unrelated primary melanomas lack genetic similarity [275]. The testing approach to establishing whether two tumors are clonally related or independent depends in part upon the clinical context and genomic features of the original tumor. There is potential for coincidental similarity in major drivers (i.e., *BRAF* V600E) between unrelated tumors, as well as clonal drift/evolution impacting non-founder mutations between related tumors. Thus, broader molecular analyses such as SNP array or large DNA panels are more useful for this purpose [277].

## 9. Molecular Testing to Distinguish Melanoma from Other Malignancies

In the context of an overtly malignant lesion, the diagnosis of melanoma can be uncertain in several scenarios. Certain other tumor types can express melanocytic markers, including sarcomas with melanocytic differentiation (e.g., clear cell sarcoma), malignant PEComa, translocation-associated renal cell carcinoma, and adrenocortical carcinoma. In melanomas without MART1 expression, the differential diagnosis might include other SOX10-positive malignancies, most notably malignant peripheral nerve sheath tumor. Although these alternative diagnostic possibilities are generally straightforward to exclude in a classic primary melanoma with an associated intraepidermal component, the distinction becomes more challenging in potential metastases of unknown primary, including cutaneous tumors that may represent metastases.

A more challenging scenario is for dedifferentiated melanoma (i.e., melanomas with areas of lost melanocytic differentiation), undifferentiated melanoma (i.e., melanomas with no demonstrated precursor or other direct evidence for melanocytic differentiation), and transdifferentiated melanoma (i.e., melanomas that display alternative differentiation such as rhabdomyosarcomatous change). In particular, undifferentiated melanoma presenting on chronically sun-damaged skin may be challenging to distinguish from atypical fibroxanthoma/pleomorphic dermal sarcoma.

Comprehensive details on the diagnostic approaches to these scenarios are highly context-dependent and beyond the scope of this review. However, a general approach includes rigorous review of clinical history (including history of malignancy elsewhere, tumor predisposition syndromes, and relevant imaging results) alongside careful morphologic assessment. An immunohistochemical panel should include both sensitive (SOX10, S100) and specific (MART1, HMB45) markers for melanocytic lineage, alongside additional markers for other malignancies on the differential diagnosis. Although not broadly specific for melanoma, PRAME can provide additional support for a diagnosis of melanoma in specific contexts such as comparisons to clear cell sarcoma or atypical fibroxanthoma/pleomorphic dermal sarcoma [278,279].

In challenging cases, molecular testing can provide additional diagnostic insights. Fusion testing may be appropriate to exclude translocation-associated tumors with melanocytic differentiation [12]. DNA-based panels can also be highly informative. UV radiation causes cyclobutane pyrimidine dimers in DNA, resulting in C>T transitions upon replication or misrepair [280]. Mutation signature analyses performed upon NGS-detected mutations determine the proportion of mutations attributable to UV radiation by comparing the tumor mutation pattern to a reference signature (e.g., COSMIC SBS7a/7b) [281,282]. UV-signature analysis may thus be helpful in differentiating cutaneous melanomas, which typically carry a UV signature, from non-melanoma mimics and from melanomas arising in sun-protected sites (e.g., acral and mucosal melanomas) [12,283]. As discussed below, methylation testing is an emerging modality for tumor classification that may also be used for this purpose.

For undifferentiated melanoma, exclusion of other undifferentiated cutaneous malignancies (atypical fibroxanthoma/pleomorphic dermal sarcoma) may be more challenging; however, differences in mutational profiles between melanoma and other cutaneous malignancies may be informative [284,285,286,287].

## 10. Emerging/Investigational Molecular Diagnostic Approaches

Beyond the established testing modalities described above, other potential avenues of molecular analysis have been explored that might eventually enter more widespread use.

Although most gene expression profiling studies have focused on protein-coding genes, differential expression of microRNAs also distinguishes melanoma from nevi [288,289,290,291,292].

Epigenetics may also offer diagnostic insights; for example, differences in DNA methylation patterns between nevi and melanoma might also be useful for this distinction, either by global profiling or focused analysis of a panel of promoters (especially *PRAME* and *CLDN11*) [293,294,295,296,297,298]. However, data on nevus subtypes remains limited, and there can be potential for challenging lesions to cluster between nevi and melanoma [297]. Although the nevus/melanoma distinction remains investigational, methylation profiling is entering routine clinical use as a tool for tumor classification, including distinguishing melanoma from other types of malignancy [299,300]; thus, the availability of this assay may facilitate expansion into other uses such as melanoma–nevus distinction.

Beyond nucleic acids, mass spectrometry analysis of cellular proteins or lipids has also been shown to distinguish nevi from melanoma [301,302,303,304,305,306]. Currently, these efforts remain investigational.

Indirect approaches to predict molecular status are also under investigation. Evidence suggests that digital image analysis of H&E-stained tumor sections may allow for rapid preliminary prediction of molecular features and malignant potential [307]. Studies on melanocytic lesions have focused on prediction of a nevus vs. melanoma diagnosis, as well as *BRAF* status [308,309,310,311,312,313,314,315,316]. In brief, although initial studies are promising, digital image analysis appears unlikely to replace more definitive molecular testing (at least in the near future), and roles for digital image analysis as a predictive screening tool in this context have yet to be defined.

## 11. Technical Considerations: Analytical Limitations in Small or Heterogeneous Specimens

Melanocytic neoplasms are frequently small, superficial, partially sampled, or inflamed and may contain significant intralesional heterogeneity (e.g., precursor nevus). These factors can further reduce the reliability of molecular assays beyond their inherent analytic limitations. Copy number assays like CGH/SNP array require at least 25% tumor purity. Similarly, NGS typically requires an analytical sensitivity of 5–10% variant allele frequency that is dependent on tumor purity. Therefore CGH/SNP array and NGS are unreliable for heavily inflamed or small lesions. Formalin fixation causes DNA fragmentation and deamination artifacts, which are worsened with prolonged fixation and storage. Furthermore, RNA is intrinsically less stable than DNA and is more vulnerable to degradation. Therefore, RNA-based assays such as GEP require even higher input and quality metrics than DNA-based assays. Mitigating strategies include microdissection to enrich tumor cells in CGH/SNP array, NGS, and GEP, visual identification of individual lesional cells in FISH, and use of recent and properly fixed and stored tissues as well as newly cut sections. In short, molecular results from small or heterogeneous melanocytic neoplasms should be interpreted in the context of tumor purity, quality metrics, and limit of detection of the assays. A negative result from a low-purity, low-quality, or partially sampled specimen should be interpreted with caution [11,12,13].

## 12. Perspectives

Profound advances in understanding of melanocytic neoplasia allow for molecular testing to provide increasingly powerful insights. However, this potential is offset by important practical considerations. Academic classification of melanocytic lesions is increasingly structured upon genomic drivers. However, molecular testing of definitively benign lesions is not justifiable from a health care resources standpoint [11]. Thus, rigorous description of morphologic features associated with molecular drivers is essential, and morphology alone must remain sufficient for clinical classification of most nevi.

For melanocytic lesions with borderline features, molecular testing can provide important contributions to diagnosis [11,317]. On this basis, NCCN Melanoma guidelines and ASDP Appropriate Use Criteria recognize the contribution of molecular testing for borderline melanocytic proliferations [317,318]. However, some test providers or insurers may require a malignant diagnosis before approving a molecular test or otherwise may consider molecular testing to not be a covered benefit. Notably, limited access to molecular testing disproportionately affects economically disadvantaged groups who are least able to cover costs out-of-pocket [319]. Resolving such access issues will be important so that all patients with challenging diagnoses can benefit from the appropriate clinical tests.

Technical limitations also impact testing availability and strategies. Detection of high-resolution genome-wide copy number profiling, DNA mutations, RNA fusions, gene expression patterns, and epigenetic alterations are currently best accomplished by distinct testing modalities [11]. Thus, complete molecular diagnostic characterization for a particular case may require multiple tests. However, the type and number of tests that can be practically completed may be limited by tumor size and purity [11].

Currently, the major roles for molecular testing in melanoma (diagnosis, prognosis, and therapeutic target identification) are often considered separately. However, there is substantial overlap between relevant molecular changes across these domains. For example, the presence of a *BRAF* V600E mutation has diagnostic significance (excludes Spitz lineage) [14,320], prognostic significance (denotes worse outcome in Spitzoid melanomas as compared to Spitz melanomas) [10,320], and therapeutic significance (supports use of BRAF/MEK inhibitor therapy for aggressive disease) [15]. As of this writing, FDA-approved systemic targeted therapies for melanoma are limited to BRAF/MEK inhibitors for *BRAF*-mutant tumors [15]; however, other oncogenic drivers in melanoma are also potentially targetable in an investigational context. As our understanding of clinically important molecular changes in melanoma continues to advance, it will be useful to consider how tests might be designed and validated to be informative across these distinct roles in order to maximize resource and tissue utilization.

Ongoing investigations will continue to improve our capabilities in molecular diagnosis of melanocytic lesions. An ideal melanocytic molecular diagnostic test would be low-cost, allow for limited sample quantity and purity, provide details on molecular drivers as well as likelihood of malignancy, and maintain high accuracy across all classes of melanocytic tumor. Future testing modalities may be more effective in meeting these needs. However, for at least the near future, a thoughtful and informed approach to melanocytic testing will remain essential for selecting the assay(s) best suited to the diagnostic questions and technical characteristics of an individual case.

## 13. Conclusion

Our understanding of the molecular basis for melanocytic neoplasia has advanced dramatically in recent years, allowing for an unprecedented precision in molecular diagnostics for challenging melanocytic lesions. FISH, array CGH, and NGS approaches can all provide insights in certain contexts. Persistent challenges relate to sample adequacy, test availability, and genomic complexity of melanocytic neoplasms. Moreover, the broad array of potential drivers and progression events can require multiple testing modalities for comprehensive characterization. Despite this added complexity, molecular testing has become increasingly valuable for improving diagnostic accuracy in challenging melanocytic cases.

## Figures and Tables

**Table 1:** Molecular Diagnostic Tests for Melanoma.

Molecular Test	Description	Positive Result (Concerning)	Negative Result	Advantages	Limitations
Melanoma FISH	Targeted evaluation for copy gains or losses	Gain of *RREB1, MYC*, or *CCND1* Single copy loss of *MYB* Homozygous loss *CDKN2A*	All probes within normal limits	Can be used for tumors with limited size, purity Allows focused evaluation of specific tumor area	No mutation or fusion data Lower sensitivity than SNP array; may be significant CNAs at loci not covered by probes Polyploidy may increase risk of false positives Loss of *MYB* may be uncertain significance in some contexts
SNP array	Genome-wide analysis for copy number and zygosity	>3 partial copy number abnormalities or any melanoma-specific gain/loss (per Alomari et al.)	Whole chromosomal changes only 3 or less partial chromosomal abnormalities (per Alomari et al.)	Well-defined algorithm for positivity	No mutation or fusion data (rearrangements may have associated CNAs) Cutoffs may require adjustment for some melanocytic neoplasms (BAP1-inactivated tumors) Requires sufficient tumor size (1 square mm) and purity Relatively greater cost
Gene expression profiling	Expression levels of a selected gene panel	Algorithmic identification of concerning expression pattern	Algorithmic identification of benign expression pattern	Well-defined algorithm for positivity	No mutation, copy number, or fusion data Independent studies have reported better performance for FISH, SNP array
Next-generation sequencing (NGS) of DNA	Mutation and copy number status of genes Targeted panel, whole exome, or whole genome	Context dependent; may include *TERT* promoter mutation, oncogene amplification, etc.	Context dependent	Identifies driver mutation if present; potential to identify therapeutic target	No fusion/rearrangement data for most panels Challenging to precisely quantitate likelihood of nevus vs. melanoma across all possible combinations of mutations (may provide more information on class than risk) Targeted panels may not include all genes of interest Copy number, heterozygosity calling less rigorous than SNP array
Next-generation sequencing (NGS) of RNA	Fusion transcript detection	Context dependent. Negative result in Spitz(oid) melanocytic lesion may suggest other driver such as *HRAS* (Spitz driver) or *BRAF, NRAS* (non-Spitz driver)	Context dependent; some fusions have stronger association with benign/malignant lesions, or may explain otherwise concerning morphology		No mutation or copy number detection Limitations vary by approach (multiplex PCR, anchored PCR, hybrid capture) Some approaches have limited or no ability to detect unexpected fusions More comprehensive approaches may be more costly and require greater tumor input

CNA: copy number alteration. FISH: fluorescence in situ hybridization. SNP: single nucleotide polymorphism. References: [11,12,13,32,176].

## Data Availability

No new data were created or analyzed in this study. Data sharing is not applicable to this article.
